# Development of a Tag/Catcher-mediated capsid virus-like particle vaccine presenting the conserved *Neisseria gonorrhoeae* SliC antigen that blocks human lysozyme

**DOI:** 10.1128/iai.00245-23

**Published:** 2023-11-02

**Authors:** Fabian G. Martinez, Ryszard A. Zielke, Cyrielle E. Fougeroux, Lixin Li, Adam F. Sander, Aleksandra E. Sikora

**Affiliations:** 1 Department of Pharmaceutical Sciences, College of Pharmacy, Oregon State University, Corvallis, Oregon, USA; 2 AdaptVac Aps, Hørsholm, Denmark; 3 Faculty of Health Science, University of Copenhagen, Copenhagen, Denmark; 4 Institute for Immunology and Microbiology, University of Copenhagen, Copenhagen, Denmark; 5 Vaccine and Gene Therapy Institute, Oregon Health & Science University, Beaverton, Oregon, USA; Universite de Geneve, Geneve, Switzerland

**Keywords:** gonorrhea, vaccine, virus-like particles, SliC, human lysozyme inhibition, Tag/Catcher-AP205 cVLP, *Neisseria gonorrhoeae*, mouse immunization

## Abstract

Virus-like particles (VLPs) are promising nanotools for the development of subunit vaccines due to high immunogenicity and safety. Herein, we explored the versatile and effective Tag/Catcher-AP205 capsid VLP (cVLP) vaccine platform to address the urgent need for the development of an effective and safe vaccine against gonorrhea. The benefits of this clinically validated cVLP platform include its ability to facilitate unidirectional, high-density display of complex/full-length antigens through an effective split-protein Tag/Catcher conjugation system. To assess this modular approach for making cVLP vaccines, we used a conserved surface lipoprotein, SliC, that contributes to the *Neisseria gonorrhoeae* defense against human lysozyme, as a model antigen. This protein was genetically fused at the N- or C-terminus to the small peptide Tag enabling their conjugation to AP205 cVLP, displaying the complementary Catcher. We determined that SliC with the N-terminal SpyTag, N-SliC, retained lysozyme-blocking activity and could be displayed at high density on cVLPs without causing aggregation. In mice, the N-SliC-VLP vaccines, adjuvanted with AddaVax or CpG, induced significantly higher antibody titers compared to controls. In contrast, similar vaccine formulations containing monomeric SliC were non-immunogenic. Accordingly, sera from N-SliC-VLP-immunized mice also had significantly higher human complement-dependent serum bactericidal activity. Furthermore, the N-SliC-VLP vaccines administered subcutaneously with an intranasal boost elicited systemic and vaginal IgG and IgA, whereas subcutaneous delivery alone failed to induce vaginal IgA. The N-SliC-VLP with CpG (10 µg/dose) induced the most significant increase in total serum IgG and IgG3 titers, vaginal IgG and IgA, and bactericidal antibodies.

## INTRODUCTION

The World Health Organization (WHO) Global Health Sector Strategy on sexually transmitted infections (STIs) notes vaccines as key innovations needed for sustainable STI control (1).

Among STI, gonorrhea is the second most reported notifiable disease in the USA after chlamydia with a total of 710,151 reported cases in 2021 (2, 3). Globally, ~82.4 million new gonorrhea infections occurred in 2020, but these statistics are underestimated due to frequent asymptomatic infections (4–6). *Neisseria gonorrhoeae* (*Ng*), the Gram-negative bacterium and etiological agent of gonorrhea, is categorized as a high-priority pathogen for research and development efforts (7–9). The Centers for Disease Control and Prevention recommends ceftriaxone for the treatment of uncomplicated gonorrhea, but failures with this therapy have occurred, and multidrug-resistant *Ng* strains are rising globally (10–18). In addition to high prevalence and antibiotic resistance, the need for developing an effective gonorrhea vaccine is exacerbated by the brunt of gonorrhea, including infertility and its ability to augment the transmission and acquisition of HIV (19). In women, gonorrhea may lead to pelvic inflammatory disease, miscarriage, preterm birth, and ectopic pregnancies. In males, this STI presents as uncomplicated urethritis but can ascend to the epididymis or testes (20). Gonorrhea primarily affects the genitourinary tract, but other mucosal surfaces can be involved, and disseminated disease may also occur (21–25). Neonatal conjunctivitis can be acquired from the infected birth canal, which if left untreated, can result in corneal scarring and blindness (25–27).

Two gonorrhea vaccines, composed of killed *Ng* and purified pilin, failed in clinical trials decades ago (28–30), illustrating the difficulty *Ng* poses to traditional vaccine design. The long-standing barriers to developing an effective *Ng* vaccine include remarkable antigenic variability, highly sophisticated strategies for modulating and evading host innate and adaptive immune responses, and the lack of established correlates of protection (31–36). To address the first challenge, we carried out proteomics and bioinformatics to identify conserved vaccine antigens (37–40). We selected the 34 gonorrhea protein antigens that were discovered through proteome-based reverse vaccinology studies and traditional approaches and carried comprehensive analyses of their sequence variation among over 5,000 clinical *Ng* isolates deposited in the *Neisseria* PubMLST database (5, 37–40). Among the most conserved antigens we identified was a *s*urface-exposed *l*ysozyme *i*nhibitor of *c*-type lysozyme, lipoprotein SliC (41). Our analysis showed that SliC is exceptionally well conserved, and over 96% of isolates have an identical SliC allele. The gene *sliC* (locus NEIP0196) has a total of 12 alleles and 22 single-nucleotide polymorphisms. There are only eight different amino acid sequences with 11 single amino acid polymorphisms distributed in <4% *Ng* isolates globally (40). In addition, utilizing Δ*sliC*, ΔsliC/p::sliC* (S83A K103A; a SliC unable to bind lysozyme), and lysozyme knockout (LysMcre) mice, we have shown experimentally that SliC provides a significant survival advantage for *Ng* during mucosal infection that is dependent on its function as a lysozyme inhibitor (41). Together, these data provide a premise for incorporating SliC in a gonorrhea vaccine.

We recognize, however, that subunit protein vaccines often fail due to low immunogenicity caused by small antigen size, instability, or improper presentation to the immune system (42, 43). Moreover, considering the mechanisms *Ng* uses to evade the human immune system, an effective vaccine may need to induce a stronger/different type of immune response compared to that elicited during infection (8, 35, 36). Subunit vaccines based on virus-like particles (VLPs) have been shown to induce potent B-cell responses in humans (44, 45), which has led to the licensure of several successful vaccines, including hepatitis B, human papillomavirus (HPV), malaria, and hepatitis E vaccines. Intriguingly, a single dose of the HPV vaccine elicited highly durable (potentially lifelong) antibody responses in humans (46). This ability is unprecedented by any other subunit vaccine and is believed to rely on the structural characteristics of the L1 antigen, which self-assembles into semi-crystalline capsid VLP (cVLP). Their antigenic similarity to virions makes them highly immunostimulatory (47). Specifically, their size (20–200 nm) and particular nature allow for passive drainage into lymph nodes, uptake by professional antigen-presenting cells, including B-cells, and innate immune system activation (48). Besides, their repetitive surface structure enables effective B-cell receptor crosslinking and B-cell activation (45, 47, 49–51). Finally, they lack genetic material and are thus non-infectious and safe. Critically for vaccine development, the intrinsic immunogenicity of cVLPs extends to protein antigens, which are displayed at high density in an orderly fashion on the cVLP (52). This is especially apparent for antigens that are otherwise weak immunogens (53, 54).

On that basis, we formulated SliC with cVLPs using the clinically validated Tag/Catcher-AP205 cVLP platform (55). The Tag/Catcher-AP205 cVLP uses a highly effective split-protein-based conjugation system, which was developed by the separation of a bacterial pilin protein into a reactive peptide (Tag) and corresponding protein-binding partner (Catcher) (42, 56). Upon mixing in the solution, the Tag and Catcher rapidly form a spontaneous isopeptide bond. This platform was developed by genetically fusing AP205 capsid to the split-protein Tag or Catcher, thus displaying 180 copies on the cVLP surface. The Tag/Catcher-AP205 has been utilized to display structurally and functionally diverse antigens, ranging in size from small peptides to large proteins (57). Importantly, the resultant VLP-displayed antigens induced antibody titers of higher quality, affinity, and avidity (42, 58–60). Recently, this vaccine technology using Tag/Catcher-AP205 decorated with the receptor-binding domain of the severe acute respiratory syndrome coronavirus 2 (SARS-CoV-2) spike protein was shown to elicit strong virus neutralization activity and is a highly promising candidate vaccine for preventing coronavirus disease 2019 (COVID-19), currently in a phase-3 clinical trial (NCT05329220) (55). The power of the Tag/Catcher-AP205 cVLP is well-documented but completely novel to the gonorrhea vaccine field (42, 45, 46, 55, 58–66).

## MATERIALS AND METHODS

### Bacteria and culture conditions

The serum-resistant *Ng* FA1090 (PorB1B) and the *Ng* 2016 WHO reference strains were used in the studies (37, 67). The ∆*sliC* knockout and complementation strain ∆*sliC/P::sliC* were constructed previously using the *Ng* FA1090 (37).

*Ng* strains were maintained on gonococcal (GC) agar [GC base (GCB); Difco] with Kellogg’s supplement I and 12.5 µM ferric nitrate or on chocolate agar plates, as indicated in the text, in a 5% CO_2_ atmosphere at 37°C for 18–20 h (68). After passage on GCB, transparent and non-piliated colonies were cultured in GCB liquid (GCBL) medium supplemented with Kellogg’s supplement I and 12.5 µM ferric nitrate (69, 70).

*Escherichia coli* strain MC1061 was utilized as a host during genetic manipulations, whereas *E. coli* BL21(DE3) was used for overproduction of rSliC, N-SliC, and C-SliC. *E. coli* strains were maintained on Luria-Bertani (LB) agar or cultured in LB broth.

Media were supplemented with antibiotics in the following concentrations: for *Ng*, kanamycin 40 µg/mL and streptomycin 100 µg/mL; and for *E. coli*, kanamycin 50 µg/mL and carbenicillin 50 µg/mL.

### Genetic manipulations

Cloning procedures, oligonucleotides, and gene blocks were designed using SnapGene software version 2.8 (GSL Biotech LLC). Primers and gene blocks were synthesized by Integrated DNA Technologies. Q5 High-Fidelity DNA polymerase, DNA ligase, and NEBuilder HiFi DNA Assembly Master Mix were obtained from New England Biolabs (NEB). All resulting genetic constructs were verified by Sanger Sequencing at the Center for Quantitative Life Sciences at Oregon State University.

To enable the fusion of a selected antigen with either SpyTagN (STN) or SpyTagC (STC), we constructed universal plasmids pET22-STN and pET22-STC, respectively, using designed *in silico* gene blocks and pET-22b(+) (EMD, Millipore). The STN DNA gene block contains a SpyTag sequence (42) followed by a linker, multicloning site, tobacco etch virus (TEV) protease cleavage site, and a 10× His Tag. The STC DNA fragment encompasses 10× His Tag followed by TEV protease recognition site, multicloning site, linker, and a SpyTag. To create pET22-STN and pET22-STC, pET-22b plasmid was amplified using primer pairs: pET-STN-forward 5′TATAGGCATCGACCATTACGATATGGGCGGCCATCGCCGGCT3′ and pET-STN-reverse 5′GAAAACCTGTACTTCCAGGGTGCCATGGATATCGGAATTAATTCGG3′ and pET-STC-forward 5′GGTGGTGGTGATGATGATGGTGGTGGGCCATCGCCGGCT3′ and pET-STC-reverse 5′CGATGCCTATAAGCCAACAAAATGAGATCCGGCTGCTAACAAAGCCC3′. The PCR products were gel-purified and assembled with corresponding gene blocks using NEBuilder HiFi DNA Assembly Master Mix.

The *sliC* gene was amplified with primers SliC-S-forward 5′GATCCCATGGCCGGAAGCGTATGATGGCG3′ and SliC-S-reverse 5′GATCCTCGAGACGGGCGCGGCAGGAAG3′ and using pRSF-SliC as a template (41). The obtained PCR product was digested with NcoI/XhoI and cloned into similarly digested pET22-STN and pET22-STC to yield pET22-N-SliC and pET22-C-SliC, respectively.

### Expression and purification of N-SliC and C-SliC

Recombinant N-SliC and C-SliC were purified from 3 L cultures of *E. coli* BL21(DE3) (71) carrying pET22-N-SliC and pET22-C-SliC, respectively. Bacteria were incubated at 37°C, 210 rpm until OD_600_ of ~0.6 was reached. Cultures were then shifted to 18°C for 1 h, and protein expression was induced with 0.1 mM IPTG. After overnight incubation, the cells were pelleted at 5,000 × *g* for 15 min at 4°C. Bacteria were suspended in binding/lysis buffer (50 mM Na_2_HPO4, pH 8, 200 mM NaCl, 25 mM imidazole, and 10% glycerol) supplemented with protease inhibitor mini tablets (Pierce) and lysed by passing through French Press at 1,500 psi. Cellular debris was removed by centrifugation at 8,000 × *g* for 15 min at 4°C, and the obtained supernatant was passaged through 0.45 µm nylon membrane (EZ flow). The cleared cell lysate was applied to a 5-mL IMAC Nickel column (Bio-Rad), and the recombinant proteins were purified on an NGC Medium-Pressure Liquid Chromatography System (Bio-Rad) using binding/lysis buffer and elution buffer (50 mM Na_2_HPO4, pH 8, 200 mM NaCl, 250 mM imidazole, and 10% glycerol). Elution fractions containing either N-SliC or C-SliC were pooled and applied onto a Vivaspin 20 centrifugal concentrator (GE HealthCare). Samples were supplemented with DTT and EDTA to final concentrations of 1 and 0.5 mM, respectively. The His Tag was removed by overnight incubation at 4°C with TEV protease in a 1:100 ratio. Dialysis was performed using a 1:4 ratio of binding/lysis buffer by placing TEV-digested samples into snakeskin dialysis tubing (3.5 MWCO) with low stirring overnight at 4°C. Samples were applied to a 5-mL IMAC Nickel column (Bio-Rad) to remove TEV. The removal of His Tag was confirmed by immunoblotting. Protein samples were concentrated as described above and subjected to size exclusion chromatography using a HiLoad 16/600 Superdex 75-pg column (GE HealthCare) with phosphate-buffered saline (PBS) as running buffer to isolate N-SliC and C-SliC. Protein purity was confirmed by sodium dodecyl sulfate–polyacrylamide gel electrophoresis (SDS-PAGE), and the protein concentration was measured using the Bio-Rad DC Protein Assay. The rSliC was purified as described previously (41).

### Expression and purification of SC-AP205 cVLPs

AP205 cVLP, complementary to N-SliC and C-SliC, was expressed in *E. coli* One Shot BL21 Star (DE3) cells and purified via an OptiPrep (Sigma) step gradient as we previously described (42, 62).

### Coupling of N-SliC and C-SliC to AP205 cVLPs

We first established optimal coupling by mixing N-SliC and C-SliC with complementary SpyCatcher VLPs at a molar ratio of 1:3 for 2 h at room temperature in PBS. The uncoupled SliC protein was removed by ultracentrifugation onto an OptiPrep step gradient and dialyzed against PBS. Subsequently, SDS-PAGE and centrifugation were used to determine the coupling efficiency (by densitometry using ImageQuant) and stability of SliC-VLP complexes (42). The quality of SliC-VLP complexes was further investigated by dynamic light scattering (DynaPro NanoStar) and negative stain transmission electron microscopy (TEM) to ensure that particles are intact and monodisperse (42, 62). For TEM, N-SliC-VLPs were adsorbed to 200-mesh carbon-coated grids, stained with 2% uranyl acetate (pH7.0), and analyzed with a Morgagni 268 electron microscope (42, 62).

### Immunization studies

Two independent immunization studies were conducted using female BALB/c mice (4–6 weeks old, Charles River Laboratories, NCI BALB/c strain). In the first study, mice (*n* = 5/group) were immunized three times in 3-week intervals subcutaneously with PBS, rSliC, or N-SliC-VLP (at 10 µg/dose; total volume of 150 µL) adjuvanted with AddaVax (InvivoGen). Retro-orbital bleeding and vaginal washes were performed on anesthetized mice (2.0% isoflurane, 1-L/m O_2_) to collect venous blood and vaginal lavages 10-day post-immunization, respectively. In an independent study, mice (*n* = 5–10/group) were administered subcutaneous (SC) vaccines (Day 0, total volume of 150 µL) containing N-SliC, N-SliC-VLP adjuvanted with AddaVax (2.5 or 10 µg/dose) and N-SliC-VLP adjuvanted with CpG ODN Class B (CpG, InvivoGen) at 2.5, 5, or 10 µg/dose followed by intranasal (IN) boost (total volume of 40 µL, given in 2, 10-µL volumes per nare, 5 min apart) on Day 21, as described previously (72). Control groups received PBS, and cVLP mixed with AddaVax or CpG (10 µg/dose). Venous blood was collected on Days 31 and 52, and vaginal lavages were collected on Day 31 as described previously (72). Blood and vaginal washes were centrifuged for 5 min, and the serum fraction and vaginal wash supernatants were stored at −20°C until further analysis.

### Enzyme-linked immunosorbent assays

Enzyme-linked immunosorbent assays (ELISAs) were performed as described previously (72) with minor modifications. Briefly, U-shaped high-binding 96-well microtiter plates (Nunc) were coated with purified N-SliC (125 ng/well) suspended in coating buffer (14 mM Na_2_CO_3_ and 35 mM NaHCO_3_) overnight at 4°C. Coated plates were blocked using Block Ace (Bio-Rad) dissolved in PBS containing 0.05% Tween-20 (PBST). Serum or vaginal lavage samples were serially diluted in PBST at varying starting dilutions (1:27 to 1:243) and added to each well. The wells were washed with PBST and incubated with diluted goat anti-mouse secondary antisera (1:10,000): total IgG, IgG1, IgG2a, IgG3, and IgA (SouthernBiotech) conjugated to horse radish peroxidase (HRP). Wells were washed, and reactions were developed using TMB Peroxidase EIA Substrate (Bio-Rad). End-point titers were determined using the average reading of eight wells incubated with secondary but no primary antibody plus 3 and 2 standard deviations as a baseline for serum and vaginal lavages, respectively. IgG1/IgG2a ratios were calculated based on group averages. For statistical analysis, Kruskal-Wallis test with Dunn’s multiple comparisons was applied to non-transformed arithmetic data. For the comparison of the two groups, the non-parametric Mann-Whitney *U* test was carried out. For all analyses, *P* values of <0.05 were considered statistically significant.

### Serum bactericidal assays

Sera from mice immunized with tested vaccines and control groups, as described in the text, were pooled and heat-inactivated for 30 min at 56°C (37, 73, 74). Subsequently, the sera were serially diluted in Hanks balanced salt solution (HBSS) at twofold dilutions starting from 1:64 to 1:8,192. The *Ng* FA1090 cells [2 × 10^4^ colony forming units (CFU/mL)] were prepared from non-piliated colonies collected from chocolate agar plates and suspended in HBSS to OD_600_ ~0.1 (1 × 10^8^ CFU/mL). The *Ng* cells (1 × 10^3^ in 40 µL) were added to wells containing test sera, mixed by shaking for 15 s, and incubated at 37°C and 5% CO_2_ atmosphere for 15 min before adding 10 µL of IgG/IgM-depleted normal human serum (NHS) or heat-inactivated NHS (HI-NHS) as the complement source (10%, vol/vol). Samples were incubated for an 1 h at 37°C with 5% CO_2_. Final suspensions (5 µL) were spot-plated onto chocolate agar plates and incubated overnight at 37°C and 5% CO_2_ for 18–20 h for CFU enumeration. Controls included *Ng* alone, *Ng* incubated with NHS (Pel-Freez), *Ng* with HI-NHS (Pel-Freez), and bacteria incubated with test sera and HI-NHS. The average percentage of killing was determined from three independent experiments by calculating the differences in the number of CFUs recovered from *Ng* incubated with test sera and NHS and the number of CFUs recovered from *Ng* incubated with test sera and HI-NHS.

### Lysozyme activity assay

To determine if the addition of SpyTag affects the SliC-mediated inhibition of c-type human lysozyme (HL, Sigma), we used the EnzChek Lysozyme Assay Kit (Thermo Fisher) as described previously (41). The lysozyme assay was carried out in black flat-bottom 96-well plates. Samples containing 2.5 µM HL (Sigma) were incubated with increasing concentrations of rSliC-STN (0–5 µM) in reaction buffer comprising of 0.1 M sodium phosphate pH 7.5, 0.1 M NaCl, and 2 mM sodium azide for 30 min at 37°C. The controls contained HL alone. After incubation, the reaction was initiated by the addition of DQ lysozyme substrate. The reaction was monitored for 20 min using a Synergy HT Microplate Reader (BioTek) at excitation and emission wavelengths of 485 and 530 nm, respectively.

To examine if immunization with N-SliC/ACP elicited antigen function-blocking antibodies, N-SliC/ACP was incubated with pooled sera from immunized and control groups (1:10, vol/vol) for 30 min and the lysozyme assays were carried out as described above.

### SDS-PAGE and immunoblotting

Samples, as indicated in the text, were standardized by OD_600_ values (whole-cell lysates) or by protein concentration and separated by SDS-PAGE on 4%–15% Bio-Rad Criterion TGX (Bio-Rad). The whole-cell lysates were prepared from Ng strains grown concurrently on solid GC medium for 20 h in 7% CO_2_ at 37°C, collected, and lysed in Laemmli SDS buffer as described previously (39, 72, 75). Proteins were visualized by Colloidal Coomassie staining or were transferred onto Trans-Blot Turbo nitrocellulose 0.2 µM membranes (Bio-Rad) using a Trans-Blot Turbo transfer system (Bio-Rad). Membranes were incubated overnight in PBST supplemented with 5% non-fat dry milk, washed with PBST, and probed with pooled sera (1:5,000) or vaginal lavages (1:50) from test or control mice followed by probing the immunoblots with goat anti-mouse IgG (Bio-Rad) or IgA (SouthernBiotech) conjugated to HRP (1:10,000 dilution) as described previously (3). Cross-reacting proteins were detected using ECL Prime (Amersham) and ImageQuant LAS 4000 (GE Healthcare).

### Statistical analysis

Statistical analyses were performed with GraphPad Prism 9 as indicated for each experimental procedure.

## RESULTS

### Design of the Tag/Catcher-AP205 platform for gonorrhea vaccine development

**Fig 1:**
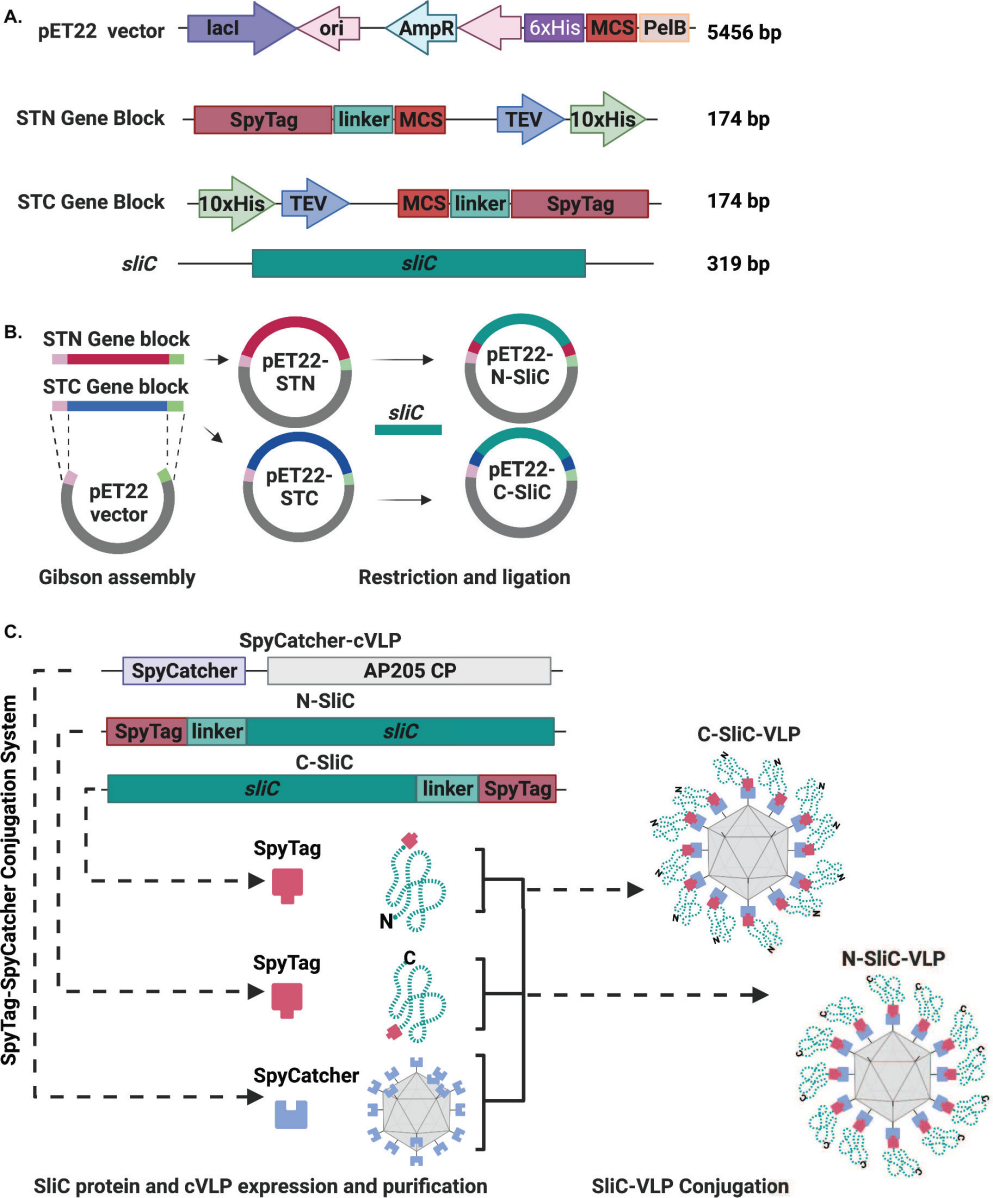
Design of the SpyTag plasmid system to explore the Tag/Catcher-AP205 cVLP platform for gonorrhea vaccine development. (**A**) An outline of *in silico* designed genetic engineering process to develop SpyTag plasmid system using pET-22b(+), *sliC* from *Ng* FA1090, and gene blocks carrying SpyTag on the N- or C-terminus (STN and STC, respectively), a linker, multicloning site, TEV protease cleavage site, and a 10× His Tag. (**B**) Gibson assembly was used to clone STN and STC gene blocks to pET-22b(+) yielding pET22-STN and pET22-STC that enable to fuse antigens with the SpyTag on either N- or C-terminus, respectively. *E. coli* PelB signal sequence is also added to promote proper antigen folding in heterologous host. The *sliC* gene was cloned into pET22-STN and pET22-STC to create pET22-N-SliC and pET22-C-SliC. (**C**) The core AP205 cVLP displaying a complementary Catcher (SpyCatcher-VLP) is expressed in *E. coli* and purified. The AP205 cVLP Catcher is mixed in solution with purified N-SliC or C-SliC. The Tag and Catcher rapidly react to form a spontaneous isopeptide bond leading to formation of N-SliC-VLP and C-SliC-VLP complexes. The figure was created with BioRender.com.

**Fig 2:**
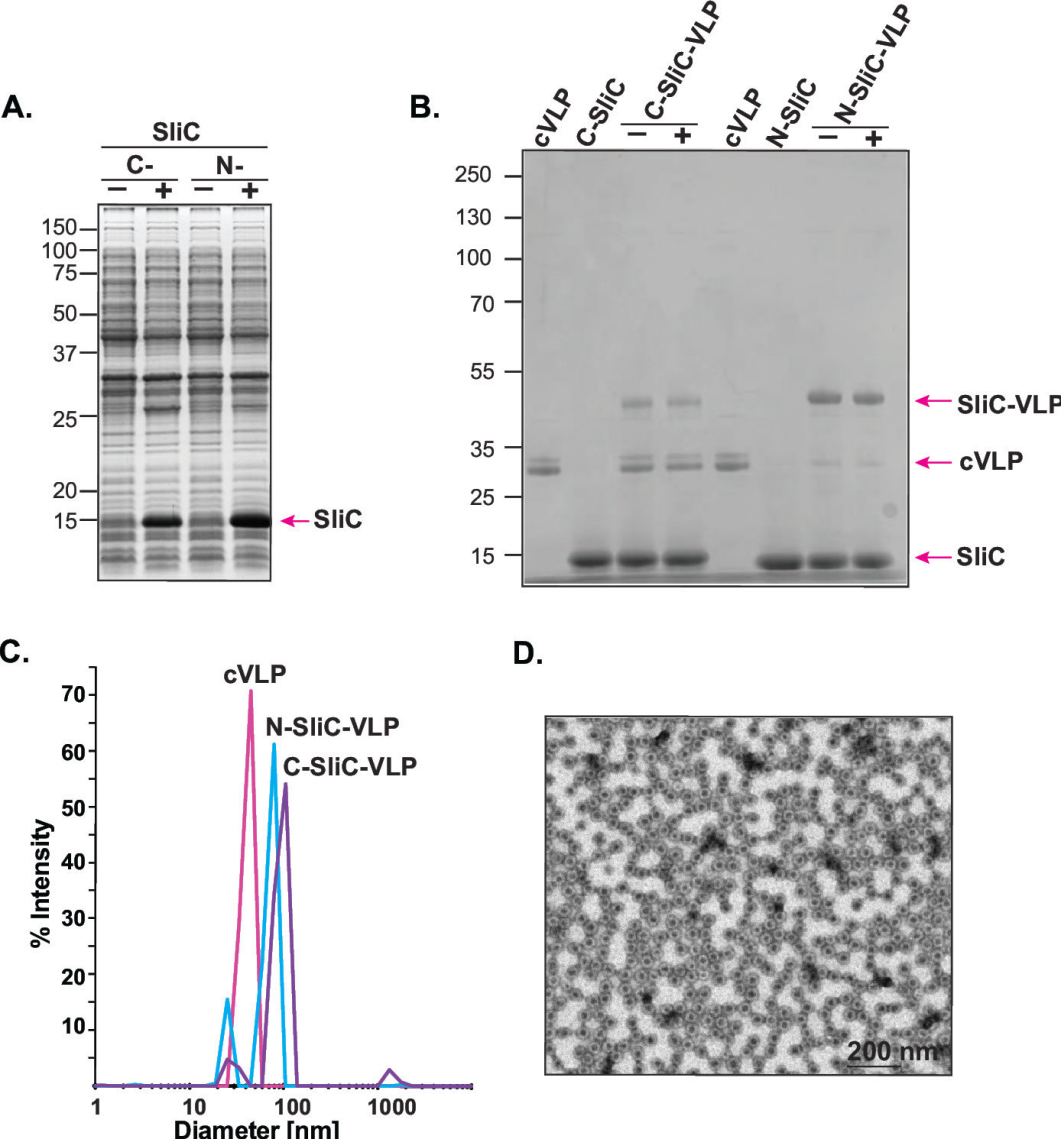
Assembly and quality assessment of the Tag/Catcher SliC-VLP vaccines. (**A**) *E. coli* BL21(DE3) carrying pET22-N-SliC and pET22-C-SliC (N and C, respectively) were cultured without (−) and with (+) IPTG. The whole-cell extracts were normalized by OD_600_, separated by SDS-PAGE, and stained with Colloidal Coomassie. Both SliC fusion proteins (N-SliC and C-SliC) were overproduced (pink arrow) and migrated on the SDS-PAGE according to the predicted molecular weight of ~15 kDa. (**B**) Purified AP205 cVLP alone (cVLP), C-SliC, N-SliC, and reaction mixtures after incubation of the cVLP and either variant of SliC (C-SliC-VLP and N-SliC-VLP) were separated by SDS-PAGE and stained with Coomassie. The covalently coupled C-SliC-VLP and N-SliC-VLP were observed together with an excess of uncoupled cVLP and SliC. Comparison of the intensity of the conjugated SliC-VLP bands before (−) and after (+) centrifugation was used to assess the aggregation state/stability of the vaccine formulation. (**C**) Dynamic light scattering of the uncoupled cVLP (in pink), the C-SliC-VLP (in lavender), and N-SliC-VLP (in blue). (**D**) N-SliC-VLPs were adsorbed to 200-mesh carbon-coated grids, stained with 2% uranyl acetate (pH7.0), and analyzed with an accelerating voltage of 80 kV, using a CM 100 BioTWIN electron microscope.

To explore the Tag/Catcher system and the *Acinetobacter* phage AP205 cVLP for GC vaccine development, we first designed gene blocks carrying SpyTag on the N- or C-terminus (STN and STC, respectively), a linker, multicloning site, TEV protease cleavage site, and a 10× His Tag (Fig. 1A) and cloned them into pET22 vector (Fig. 1B). This newly engineered pET22-STN and pET22-STC system enables cloning and production of a selected antigen with the SpyTag placed on either N-terminus or C-terminus to ensure optimal antigen folding for purification and coupling to the cVLP (Fig. 1C). In each case, an *E. coli* PelB signal sequence is also added to promote proper antigen folding in heterologous host. We selected the SliC antigen from *Ng* FA1090, a strain that carries the most broadly distributed SliC antigen variant (40), and cloned *sliC* (NGO1063) into pET22-STN and pET22-STC (Fig. 1B). Both SliC fusion proteins with SpyTag positioned on the N or C terminus, N-SliC or C-SliC, respectively, were successfully overproduced in *E. coli* and migrated on the SDS-PAGE according to the predicted molecular weight of ~15 kDa (Fig. 2A). Highly pure N-SliC and C-SliC were obtained via affinity chromatography steps, removal of cleavable His_10_-tag by TEV protease, followed by size exclusion chromatography (Fig. 2B, Lanes 2 and 6).

### Assembly of the Tag/Catcher SliC-VLP vaccines

To generate Tag/Catcher SliC-VLP vaccines, individual N-SliC and C-SliC proteins were combined with the AP205 capsid protein carrying the complementary Catcher (Fig. 1D and 2B). Quality assessment of the two different SliC-VLP complexes by SDS-PAGE revealed the covalently coupled C-SliC-VLP and N-SliC-VLP (migrating at ~45 kDa) along with uncoupled cVLP, SliC-STC, and SliC-STN (Fig. 2B). Excess amounts of uncoupled cVLP were observed in a reaction containing C-SliC as compared to the reaction containing N-SliC. Additionally, the amount of coupled C-SliC-VLP was lower in comparison to the N-SliC-VLP (Fig. 2B). These results revealed that N-SliC coupled onto the cVLP to a higher efficiency. Albeit there was similar intensity between C-SliC-VLP and N-SliC-VLP bands before (−) and after (+) centrifugation, demonstrating that both vaccine formulations are stable. Dynamic light scattering of the uncoupled cVLP-Catcher, C-SliC-VLP, and N-SliC-VLP showed that all samples are monodisperse with a peak around the expected particle size (30–40 nm), indicating that the core cVLP and SliC-VLP complexes are not aggregated. As expected, coupling of SliC antigen to the cVLP caused a slight increase in the diameter of the particles of 47.7 and 36.9 nm for C-SliC-VLP and N-SliC-VLP, respectively, compared to 20.8 nm of cVLP (Fig. 2C). The C-SliC-VLP had a higher polydispersity (28.7%) compared to N-SliC-VLP (11.7%) and cVLP alone (12.1%), indicating that the C-SliC-VLP population is more heterogeneous and that the coupling is not optimal. Due to the higher coupling efficiency (77%) with 140 subunits out of 180 per cVLP carrying the SliC antigen, we selected N-SliC-VLP for further studies. TEM confirmed that N-SliC-VLP contains intact and monodisperse particles (Fig. 2D). To evaluate whether the addition of STN affects SliC inhibitory activity of HL, we performed titration reactions with increasing concentrations of purified N-SliC in the presence of peptidoglycan (Fig. S1A). Similar to untagged, recombinant SliC (41), N-SliC inhibited the lytic activity of HL in a dose-dependent manner, with complete blocking of lysozyme function at concentrations above 1.25 µM.

These evaluations showed that N-SliC antigen is functional and couples more efficiently to the cVLPs, and thus, vaccine formulation containing N-SliC-VLP was selected for further immunization studies.

### cVLP significantly enhances SliC immunogenicity and serum bactericidal activity and promotes a Th1 response

To assess SliC as a gonorrhea vaccine antigen and the impact of presenting SliC on the cVLP, we immunized mice with N-SliC-VLP, N-SliC, or cVLP using three SC injections at 3-week intervals. Balanced Th1/Th2 responses are considered optimal for some vaccines (76), and therefore, in the first assessment of SliC as an antigen, we adjuvanted all treatments with AddaVax—a squalene-based oil-in-water emulsion formulation similar to that of MF59, which is licensed in Europe for flu vaccines—which elicits both cellular Th1 and humoral Th2 responses (77).

**Fig 3:**
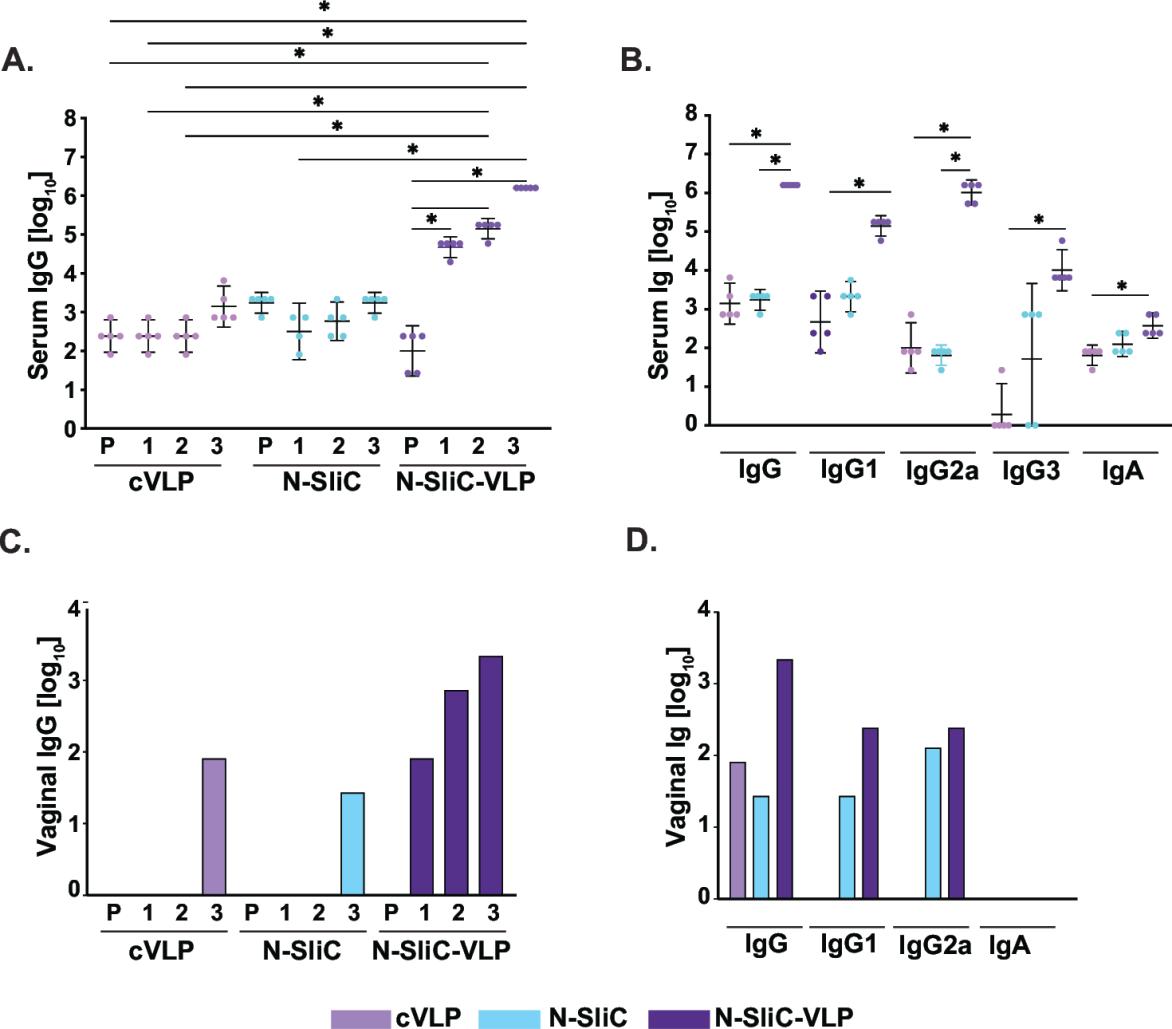
The N-SliC-VLP vaccine adjuvanted with AddaVax markedly induced antibody titers compared to corresponding vaccine containing monomeric N-SliC. (**A**) Systemic total IgG titers were examined in pre-immune (P) and 10 days after first (1), second (2), and third (3) subcutaneous administration of cVLP, N-SliC, or N-SliC-VLP (*n* = 5 mice/group). All treatments were adjuvanted with AddaVax. (**B**) Total IgG, IgG1, IgG2a, IgG3, and IgA antibody titers in final sera from mice immunized with cVLP, N-SliC, or N-SliC-VLP. (**C**) Vaginal total IgG titers in pooled vaginal washes were examined in pre-immune (P) and 10 days after first (1), second (2), and third (3) subcutaneous administration of cVLP, N-SliC, or N-SliC-VLP. (**D**) Total IgG, IgG1, IgG2a, and IgA titers in final pooled vaginal lavages obtained from mice immunized with cVLP, N-SliC, or N-SliC-VLP. Bar graphs represent geometric mean ELISA titers against N-SliC with error bars showing 95% confidence limits. Statistical significance was determined using Kruskal-Wallis with Dunn’s multiple comparison test. For the comparison of two groups, the non-parametric Mann-Whitney *U* test was carried out. For all analyses, *P < 0.05.

ELISA performed on samples after each immunization showed a substantial rise in serum total IgG in mice administered with N-SliC-VLP compared to the corresponding total IgG detected in mice administered with N-SliC and VLP (Fig. 3A). Additionally, each boost of the N-SliC-VLP vaccine led to a significant increase in IgG antibody. The levels of total IgG detected after immunization with N-SliC and cVLP were comparable to the total IgG detected in pre-immune sera (Fig. 3A). The calculated geometric mean of total IgG antibody responses in terminal sera from mice immunized with N-SliC-VLP was 1.6 × 10^6^ compared to 1.7 × 10^3^ and 1.4 × 10^3^ in mice that received N-SliC and cVLP, respectively (Fig. 3B). Furthermore, the N-SliC-VLP vaccine elicited markedly increased IgG1, IgG2a, IgG3, and IgA titers than N-SliC or cVLP (Fig. 3B). Immunization with N-SliC-VLP resulted in boost of IgG2a and IgG1 antibody responses (1.03 × 10^6^ and 1.4 × 10^5^, respectively) that were significantly greater than with N-SliC (0.06 × 10^3^ and 2.1 × 10^3^, respectively) or cVLP (0.1 × 10^3^ and 0.5 × 10^3^, respectively). The IgG1/IgG2a ratio in mice given N-SliC-VLP (0.14) suggested a systemic Th1 bias. Serum IgG3 titers, predictive of serum bactericidal assay (SBA) activity (78), were 168- and >10,000-fold higher in N-SliC-VLP-immunized mice than in N-SliC and cVLP groups, respectively (Fig. 3B). ELISA of pooled vaginal lavages within a group showed that mice administered with N-SliC-VLP vaccine had increased vaginal IgG after the first immunization that augmented with each boost, whereas mice that received N-SliC or cVLP had undetectable IgG in the first and second vaginal samples and ~100-fold lower titers in the terminal vaginal lavages (Fig. 3C). Vaginal IgG, IgG1, and IgG2a were greater after the final immunization of N-SliC-VLP vaccine with the titers of 2.2 × 10^3^, 0.243 × 10^3^, and 0.243 × 10^3^, respectively, in comparison to N-SliC (0.027 × 10^3^, 0.027 × 10^3^, and 0.127 × 10^3^) and cVLP (0.081 × 10^3^, not detectable); however, IgA antibody subtypes were not detected (Fig. 3C and D).

**Fig 4:**
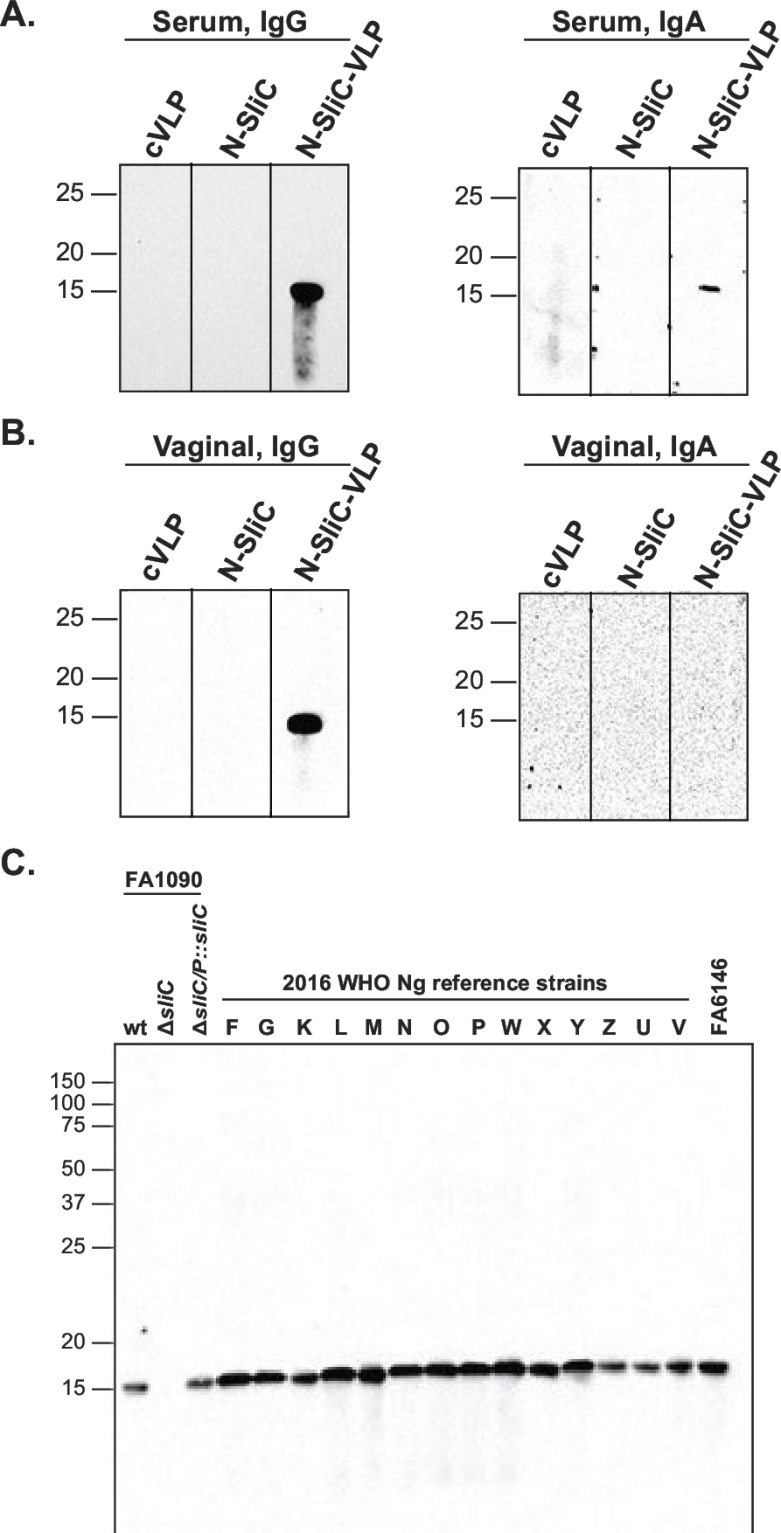
SliC-specific systemic IgG and IgA and vaginal IgG were elicited by subcutaneous immunization with N-SliC-VLP vaccine adjuvanted with AddaVax. Female mice (*n* = 5) were subcutaneously immunized with cVLP, N-SliC, or N-SliC-VLP adjuvanted with AddaVax. Purified N-SliC (**A and B**) and whole-cell extracts obtained from the isogenic *Ng* strain FA1090, the Δ*sliC* knockout, the complemented Δ*sliC*/P::*sliC*, and a panel of geographically, genetically, and temporally diverse *Ng* isolates were fractionated by SDS-PAGE. Immunoblotting was performed with pooled serum (**A and C**) and vaginal washes (**B**) collected after the third immunization, followed by secondary antibodies against mouse IgG (**A and C**) or IgA (**A and B**).

To examine the specificity of immune responses elicited by the N-SliC and N-SliC-VLP vaccines, we performed an immunoblotting analysis with either purified N-SliC or a panel of whole-cell extracts obtained from different *Ng* strains including the 2016 WHO reference isolates (Fig. 4). SliC-specific IgG and IgA were detected in serum from mice immunized with N-SliC-VLP (Fig. 4A and B, respectively), while in contrast, no signal was observed after blotting the membranes with serum or vaginal washes from N-SliC or cVLP-immunized mice. Vaginal IgG but not IgA obtained from mice that received N-SliC-VLP readily recognized purified N-SliC (Fig. 4B). Furthermore, the N-SliC-VLP vaccine elicited systemic IgG that recognized native SliC in the isogenic strain FA1090, the complemented Δ*sliC*/P::*sliC*, and, importantly, in the 14 diverse strains of the 2016 panel of WHO isolates and *Ng* FA6146. As expected, no signal was detected in the Δ*sliC* knockout strain (Fig. 4C).

Finally, we sought to assess whether SliC-containing vaccines induce functional antibodies by examining serum bactericidal killing (SBA) and interference with SliC function as a lysozyme inhibitor (41). We used pooled murine sera from each examined group in combination with IgG- and IgM-depleted NHS to assess SBA titers against the serum-resistant *Ng* FA1090 (37, 73, 74). Human complement-dependent SBA titer (50% *Ng* killing) was greater than or equal to fourfold in mice that received the N-SliC-VLP vaccine compared to N-SliC and cVLP groups, respectively (Table 1).

**TABLE 1:** Human serum bactericidal activity of pooled murine antisera to N-SliC and N-SliC-VLP vaccines delivered with different adjuvants^*a*^

Antigen	Adjuvant	Immunization route	SBA titer against *Ng* FA1090
cVLP	AddaVax	3× SC	1,024 (512, 1,024, 1,024)
N-SliC	AddaVax	3× SC	1,024 (1,024, 1,024, 2,048)
N-SliC-VLP	AddaVax	3× SC	4,096 (4,096, 4,096, 4,096)
No antigen	Saline	SC, IN	512 (512, 512, 1,024)
N-SliC^*b*^	Saline	SC, IN	512 (512, 512, 1,024)
cVLP^*b*^	AddaVax	SC, IN	512 (512, 512, 512)
cVLP^*b*^	CpG	SC, IN	1,024 (1,024, 1,024, 1,024)
N-SliC-VLP^*b*^	AddaVax	SC, IN	512 (512, 512, 1,024)
N-SliC-VLP^*b*^	CpG	SC, IN	4,096 (4,096, 4,096, 4,096)

^*a*^ Pooled antisera obtained from mice groups that were immunized as outlined above and, in the text, and the corresponding sham-immunized controls were tested for their ability to induce SBA killing of *Ng* FA1090. The data represent the reciprocals of the highest serum dilution at which ≥50% killing was noted. The titers are expressed as the median values from biological replicate experiments (*n* = 3), and the values in parentheses designate the SBA titers.

^*b*^ Formulations were administered at 10 µg/dose/mouse.

To evaluate if the anti-SliC antibody interferes with SliC function, we examined HL activity in the presence of purified SliC using a fluorescein-labeled peptidoglycan (41, 79). The lytic activity of HL remained blocked in the presence of SliC regardless of whether the antigen was pre-incubated with decomplemented sera obtained from mice immunized with N-SliC-VLP, N-SliC, or cVLP (Fig. S1C). In contrast, rabbit sera against another *Ng* lysozyme inhibitor, ACP, restored lysozyme activity, confirming that ACP elicits functional blocking antibodies (80) whereas SliC remained active against HL in the presence of rabbit anti-SliC (Fig. S1B and C).

Cumulatively, these results showed that while monomeric SliC is not immunogenic in mice, our cVLP platform markedly improves antigen immunogenicity and antibody kinetics, elicits bactericidal antibodies, and induces a potentially protective Th1-biased response against *Ng* (81–86).

### SC and IN administration of N-SliC-VLP vaccine formulated with AddaVax or CpG

To promote both systemic and mucosal immune responses, our next choice for the immunization route was a SC prime followed by IN boost. Administration of the MetQ-CpG vaccine in a similar manner induced a protective immune response and IgA at the vaginal mucosae (81). We also sought to examine the impact of vaccine dosage (2.5–10 µg/dose) and formulation on antibody responses. In addition to AddaVax, we adjuvanted the N-SliC-VLP vaccine with CpG ODN based on the enhanced *Ng* clearance from the murine lower genital tract after immunization with antigens administered with Th1-inducing adjuvants including MetQ-CpG, MtrE-CpG, the lipooligosaccharide (LOS) 2C7 epitope mimic with MPLA, and *Ng* OMVs-IL-12 or viral replicon particles with PorB (81–86).

**Fig 5:**
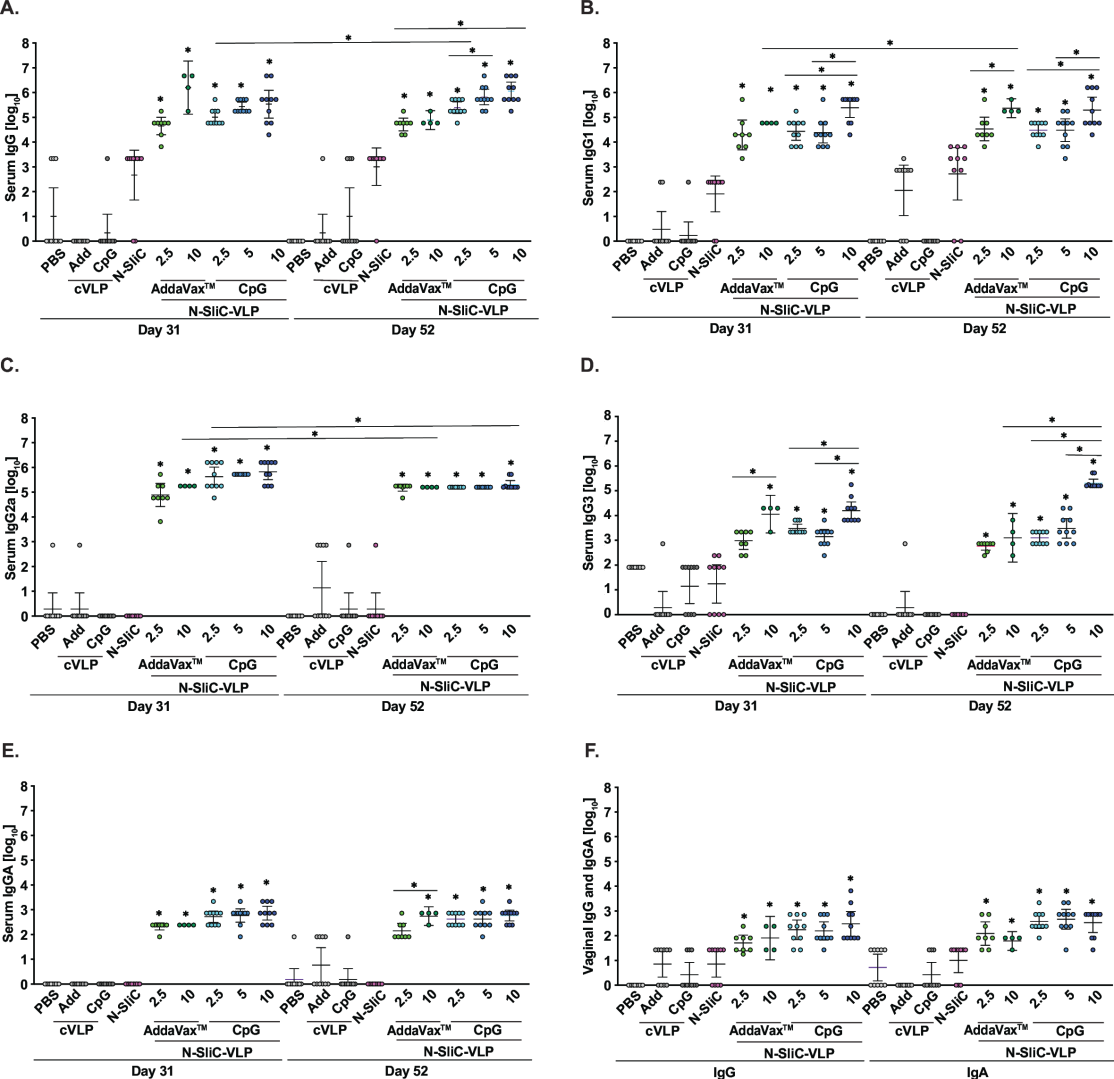
Anti-SliC antibody titers elicited by N-SliC and N-SliC-VLP subcutaneous and intranasal immunization. Post-immunization (Days 31 and 52) total IgG (**A**), IgG1 (**B**), IgG2a (**C**), IgG3 (**D**), and IgA (**E**) antibody titers in sera from mice (*n* = 5–10 mice/group) immunized with N-SliC-VLP-AddaVax (2.5 and 5 µg/dose), N-SliC-VLP-CpG (2.5, 5, and 10 µg/dose), N-SliC, VLP-AddaVax, cVLP-CpG, or unimmunized (PBS). (**F**) Post-immunization (Day 32) vaginal IgG and IgA titers were assessed in female mice administered with N-SliC-VLP-AddaVax (2.5 and 5 µg/dose), N-SliC-VLP-CpG (2.5, 5, and 10 µg/dose), N-SliC, cVLP-AddaVax, cVLP-CpG, or PBS. Bar graphs represent geometric mean ELISA titers with error bars showing 95% confidence limits. Statistical significance between data in groups was determined using Kruskal-Wallis with Dunn’s multiple comparison test. For the comparison of two groups, the non-parametric Mann-Whitney *U* test was carried out. *P < 0.05.

Following the immunizations, the immune responses to N-SliC, N-SliC-VLP-AddaVax, and N-SliC-VLP-CpG vaccines were examined by assessing the reactivity of individual murine antisera against purified N-SliC in ELISA (Fig. 5). There was a significant increase in systemic antibody titers at the two data points (Days 31 and 52) for total IgG, IgG1, IgG2a, IgG3, and IgA in all N-SliC-VLP vaccine experimental groups regardless of vaccine dose in comparison to the control groups that received PBS, cVLP- AddaVax, or cVLP-CpG (Fig. 5A through D). Similarly, to our previous study (Fig. 3 and 4), N-SliC failed to induce significant antibody titers although administration of N-SliC led to 1,000- to 100-fold rise in the SliC-specific IgG (1.01 × 10^4^ geometric mean, final sera) in comparison to controls (0.001 × 10^3^ for PBS, 0.002 × 10^3^ for cVLP- AddaVax, and 0.01 × 10^3^ for cVLP-CpG; Fig. 5A). Titers were insignificant and negligible for IgG1, IgG2a, IgG3, and IgA (Fig. 5B through E, respectively). Importantly, our vaccine dose studies showed that the N-SliC-VLP-CpG (10 µg/dose) elicited the highest total IgG titers in the final sera with the geometric mean of 1.15 × 10^7^, which was 4.6-, 1.7-, 22-, and 14.7-fold greater than the titers induced by the same vaccine at 2.5 and 5 µg/dose, and the N-SliC-VLP-AddaVax at 2.5 and 10 µg/dose, respectively (Fig. 5A). The serum IgG1 levels were the greatest in mice that received N-SliC-VLP at 10 µg/dose formulated with AddaVax or CpG and reached 2.3 × 10^5^ and 1.98 × 10^5^, respectively (Fig. 5B). Statistical analyses revealed no significant differences in IgG2a titers in murine sera between the different vaccine groups; however, the titers slightly declined in final sera in mice administered with vaccines formulated with CpG in comparison to the respective samples obtained on Day 31 (Fig. 5C). The IgG1/IgG2a ratios were 0.22, 1.46, and 0.19, 0.19, and 0.96 in mice that received N-SliC-VLP-AddaVax at 2.5 and 5 µg/dose and N-SliC-VLP-CpG at 2.5, 5, and 10 µg/dose, respectively. These results suggest that the highest tested N-SliC-VLP vaccine doses formulated with AddaVax/CpG elicited more balanced Th1/Th2 immune responses, whereas the lower doses induced Th1-biased immune responses. Furthermore, N-SliC-VLP-CpG at 10 µg/dose resulted in the greatest IgG3 antibody titers in comparison to all tested vaccine formulations with the geometric mean of 2.05 × 10^5^ that increased 67-, 162-, and 354-fold in comparison to those in mice administered with 5 µg of N-SliC-VLP-CpG, 2.5 µg of N-SliC-VLP-CpG/10 µg of N-SliC-VLP-AddaVax, and 2.5 µg of N-SliC-VLP-AddaVax, respectively (Fig. 5D). Mice immunized with both N-SliC-VLP-AddaVax and N-SliC-VLP-CpG elicited similar systemic IgA responses (Fig. 5E) with the titers of 0.14 × 10^3^ (2.5 µg of N-SliC-VLP-AddaVax), 0.55 × 10^3^ (10 µg of N-SliC-VLP-AddaVax), 0.42 × 10^3^ (2.5 µg of N-SliC-VLP-CpG), 0.42 × 10^3^ (5 µg of N-SliC-VLP-CpG), and 0.58 × 10^3^ (10 µg of N-SliC-VLP-CpG).

**Fig 6:**
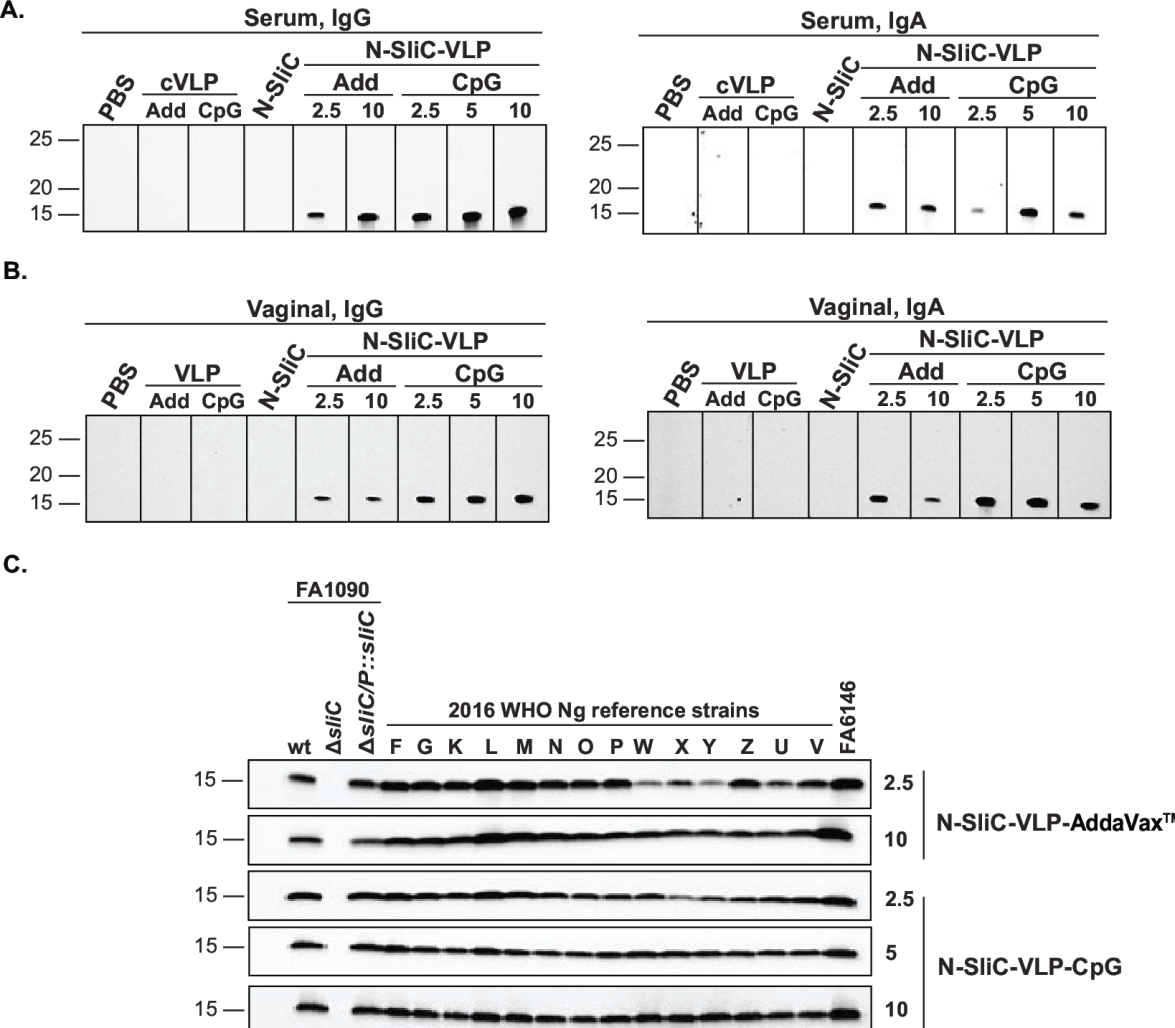
N-SliC-VLP-AddaVax/CpG vaccines elicited SliC-specific systemic and vaginal IgG and IgA after subcutaneous immunization and intranasal boost. Female mice (*n* = 5–10 mice/group) were administered with N-SliC-VLP-AddaVax (Add; 2.5 and 5 µg/dose), N-SliC-VLP-CpG (2.5, 5, and 10 µg/dose), N-SliC, cVLP-AddaVax (cVLP-Add), cVLP-CpG, or PBS, as indicated. Purified N-SliC (**A and B**) and whole-cell extracts obtained from the isogenic *Ng* strain FA1090, the Δ*sliC* knockout, the complemented Δ*sliC*/P::*sliC*, the 2016 WHO *Ng* panel, and FA6146 were separated by SDS-PAGE. Immunoblotting was performed with murine pooled serum (**A and C**) and vaginal washes (**B**) collected after the second immunization, followed by secondary antibodies against mouse IgG (**A and C**) or IgA (**A and B**).

As expected from previous investigations that used the mucosal immunization route (81, 87), our ELISA experiments showed a significant increase in titers of IgG and IgA in vaginal lavages in mice administered with all vaccine formulations in comparison to SliC alone and all control groups (Fig. 5F). Corroborating the ELISA findings, SliC-specific IgG and IgA were detected in serum and vaginal lavages derived from mice administered with N-SliC-VLP vaccines formulated with AddaVax/CpG as shown by immunoblotting in Fig. 6A and B, respectively. Furthermore, all vaccine doses and formulations elicited systemic IgG that cross-reacted with SliC protein in whole-cell extracts of the 2016 panel of *Ng* WHO isolates and FA6146 (Fig. 6C). Specificity of the elicited antibodies was apparent by the absence of reactivity of any of the anti-SliC sera with the corresponding *Ng* FA1090 Δ*sliC* strain and the lack of reactivity of PBS- and cVLP-AddaVax/CpG-immunized sera with SliC in isogenic wild-type bacteria and the complemented Δ*sliC*/P::*sliC* (Fig. 6).

On the basis of the IgG3 titers (Fig. 5D), we performed the human complement-dependent SBA with pooled murine sera obtained from animals immunized with the N-SliC-VLP-CpG/AddaVax vaccines at 10 µg/dose. Reciprocal sera dilutions at which 50% *Ng* killing was observed were fourfold to eightfold higher for N-SliC-VLP-CpG in comparison to bactericidal titers generated by immunization with N-SliC, controls (PBS, cVLP-CpG/AddaVax), and N-SliC-VLP-AddaVax (Table 1). The lack of SBA activity in mice that received N-SliC-VLP-AddaVax could be attributed to several factors including different immunization routes and dosing schedule, as well as waning IgG3 titers. Indeed, sera from mice immunized intramuscularly with NspA vaccine had the strongest bactericidal activity against *Ng* followed by sera from the IN and intravaginal immunization cohorts (88). Furthermore, in humans, detergent-extracted meningococcal OMVs vaccine induced significantly lower SBA levels when administered IN than via intramuscular route (89).

Finally, we assessed whether vaccination with N-SliC-VLP-AddaVax/CpG given via SC and IN route induces antibodies that block the SliC function as a lysozyme inhibitor (Fig. S1D). Similar to previous immunization studies (Fig. S1C), the lytic activity of HL against peptidoglycan was blocked in the presence of purified SliC irrespective of the presence of anti-SliC sera (Fig. S1D).

Together, these studies showed that N-SliC-VLP-AddaVax/CpG administered SC followed by IN boost, induced SliC-specific systemic and mucosal IgG and IgA. Furthermore, IgG1/IgG2a ratios depended on vaccine dosage with the lower doses (2.5–5 µg) corresponding to bias toward Th1 response and the higher doses (10 µg) eliciting a more balanced Th1/Th2 response. The higher vaccine doses resulted in greater IgG1 and IgG3 titers. The data suggest that the N-SliC-VLP-CpG (10 µg/dose) is a promising vaccine for follow-up studies due to its ability to induce the most significant increase in total serum IgG and IgG3 titers and functional antibodies with SBA activity.

## DISCUSSION

VLPs have gained mounting recognition as promising nanotools for vaccine development due to their safety and ability to provide strong and long-lasting immune responses even after one administration (51, 90, 91). Several VLP vaccines have been developed and commercialized: Recombivax (Merck) and Engerix (GlaxoSmithKline) for hepatitis B (92, 93), Hecolin (Innovax) for hepatitis E (94), and more relatedly to STDs, Gardasil (Merck), and Cervarix (GlaxoSmithKline) for HPV (45, 95, 96). Clinically tested VLP vaccines have been extended to influenza A, chikungunya virus, human cytomegalovirus, and human norovirus and in SARS-CoV-2 using the AP205 platform (52, 97, 98). Protein and peptide antigens are frequently displayed on VLPs either through the genetic fusion of epitopes to the self-assembling coat protein or through chemical conjugation to the surface of pre-assembled VLPs. These strategies have their drawbacks, for example, limitation on antigen size, low-density coupling, interference with VLP assembly, and narrow, epitope-specific antibody responses (45, 53, 58). The Tag/Catcher-AP205 cVLP platform composed of peptide counterparts SpyTag and SpyCatcher that form irreversible spontaneous isopeptide bond circumvent these challenges (56). The *Acinetobacter* phage AP205 is an attractive VLP vaccine backbone due to its unique structure in that both the N- and C- termini are surface exposed and evenly distributed on the assembled VLP, allowing for genetic fusions at both termini while maintaining stable assembly. Additionally, AP205 is an attractive VLP vaccine backbone due to intrinsic immunogenicity, lack of pre-existing immunity in humans, manufacturability, and cost-effective production in *E. coli* (42, 45, 99–101).

Currently, no licensed gonorrhea vaccines exist, and the foci of attention in the field are on outer membrane vesicles, protein subunit vaccines, and a peptide mimic of a glycan epitope of *Ng* LOS 2C7 as vaccine candidates (102). As discussed above, protein subunit vaccines can greatly benefit from VLP display, but, to date, this platform has not been explored in the gonorrhea field. Herein, we showed the premise of this approach by using the Tag/Catcher-AP205 cVLP for delivery of a novel gonorrhea antigen SliC, testing different vaccine formulations, doses, and immunization routes. SliC is a remarkably conserved surface lipoprotein that contributes to bacterial defense against the host innate immune system effector, lysozyme (41). We designed and engineered two plasmids that enable expression and purification of different *Ng* antigens fused with SpyTag on the N- or C-terminus (Fig. 1). Furthermore, we determined that both N- and C-SliC could be efficiently overproduced and purified; however, the C-terminal SpyTag hampered the assembling efficiency of SliC to VLPs (Fig. 2A and B, respectively). In contrast, the N-SliC antigen variant readily loaded onto AP-205 cVLP forming stable multivalent complexes and retained its function as an HL inhibitor (Fig. 2B through D; Fig. S1B, respectively).

Our investigations showed that vaccines containing monomeric N-SliC failed to induce SliC-specific antibodies when administered alone/AddaVax via different immunization routes in mice (Fig. 3 to 6). The recombinant SliC protein emulsified in Freund’s complete adjuvant and administered SC elicited immune responses in rabbits that recognized the purified SliC variant and native SliC in diverse panel of *Ng* isolates (41). The limited immunogenicity displayed by SliC in mice could be caused by loss of its conformational stability in PBS/AddaVax formulation that affects antigen processing in antigen-processing cells from its uptake to the final presentation of antigenic peptides. Destabilization of a protein’s three-dimensional structure can lead to the loss of individual epitopes or redirection of antibody responses to irrelevant epitopes that are not favored in the wild-type conformation. The presence of adjuvants may affect protein folding and even lead to protein denaturation (103). In contrast to monomeric N-SliC vaccines, the multivalent repetitive and particulate display of N-SliC via the Tag/Catcher-AP205 cVLP (Fig. 2) significantly potentiated its immunogenicity as shown by increased kinetic of antibody responses markedly induced antibody titers in ELISA, serum and vaginal SliC-specific IgG and/or IgA, and functional antibodies with SBA activity (Fig. 3 to 6; Table 1).

The ability of *Ng* to evade adaptive immune responses and the lack of established correlates/mechanisms of protection in mice or humans are long-standing barriers in the gonorrhea vaccine field (35). Work by Liu et al. has suggested that Th1 responses are important for the efficacy of an intravaginal vaccine formulation composed of *Ng* OMVs in combination with encapsulated poly-lactic acid microspheres containing IL-12, a Th1-inducing cytokine (104). Our data suggest that the dose and delivery route of the N-SliC-VLP vaccine affect Th1 and Th2 responses as assessed by IgG1/IgG2a ratios (Fig. 3 and 5). The N-SliC-VLP-AddaVax administered via 3× SC elicited Th1-biased immune response whereas the same vaccine formulation and dose delivered by SC, IN routes induced Th1/Th2-balanced responses. Similarly, PorB DNA-based vaccine induced both Th1 and Th2 responses, depending on the method of delivery (105). Although the function of antibodies in protection against *Ng* is not fully elucidated, several lines of evidence suggest that antibodies may play a crucial role. In addition to Th1 responses, the *Ng* OMVs-IL-12 vaccine depends on B-cell production of antibodies (85). The efficacy of the LOS mimitope-based vaccine (TMCP2) requires IgG and complement (106), as does the efficacy of monoclonal antibodies against this LOS 2C7 epitope vaccine requires complement (73). IgG is crucial for many effector functions of bacterial vaccines, including the blocking of function (e.g., tetanus-diphtheria vaccine) and through direct SBA (e.g., *Neisseria meningitidis*) (107, 108). Unlike the gastrointestinal tract, where IgA predominates, the IgG is the most abundant Ig in the female genital tract (109). Passively administered antibodies protect against HPV (108). Serum IgG, as generated by systemic immunization, is readily transported into the genital tract through FcRn (110). GC infections primarily localize to mucosal surfaces; thus, boost of mucosal IgA may be important for protection against *Ng* infection. Indeed, MetQ-CpG and *Ng* OMVs-IL-12 administered via IN route elicited vaginal IgA and accelerated *Ng* clearance from the murine lower genital tract (72, 87).

We conclude that the N-SliC-VLP-CpG (10 µg/dose) administered via SC and IN is a promising vaccine for follow-up studies due to its ability to elicit systemic and mucosal IgG and IgA, boost the serum IgG and IgG3 responses, and induce functional antibodies with SBA activity.

## SUPPLEMENTAL MATERIAL

Figure S1 iai.00245-23-s0001.pdf Lysozyme assay.

ASM does not own the copyrights to Supplemental Material that may be linked to, or accessed through, an article. The authors have granted ASM a non-exclusive, world-wide license to publish the Supplemental Material files. Please contact the corresponding author directly for reuse.

## References

[1] Organization WH. 2016. Global health sector strategy on sexually transmitted infections, 2016–2021

[2] CDC. 2018. Sexually transmitted diseases surveillance: CDC.Gov; 2019 [updated 2019-10-09T12:34:07Z]. Available from: https://www.cdc.gov/std/stats18/default.htm

[3] Prevention CfDCa. 2021. Sexually transmitted disease surveillance, 2021. Available from: https://www.cdc.gov/std/statistics/2021/default.htm

[4] Rowley J, Vander Hoorn S, Korenromp E, Low N, Unemo M, Abu-Raddad LJ, Chico RM, Smolak A, Newman L, Gottlieb S, Thwin SS, Broutet N, Taylor MM. 2019. Chlamydia, gonorrhoea, trichomoniasis and syphilis: global prevalence and incidence estimates, 2016. Bull World Health Organ 97:548P–562P. doi:10.2471/BLT.18.228486 31384073 PMC6653813

[5] Rice PA, Shafer WM, Ram S, Jerse AE. 2017. Neisseria gonorrhoeae: drug resistance, mouse models, and vaccine development. Annu Rev Microbiol 71:665–686. doi:10.1146/annurev-micro-090816-093530 28886683

[6] Organization WH. 2023. Gonorrhoea (Neisseria gonorrhoeae infection). Available from: https://www.who.int/news-room/fact-sheets/detail/gonorrhoea-(neisseria-gonorrhoeae-infection)

[7] Organization WH. 2017. Global priority list of antibiotic-resistant bacteria to guide research, discovery, and development of new antibiotics; 2017 [updated 2017/02/27/]

[8] Gottlieb SL, Ndowa F, Hook EW, Deal C, Bachmann L, Abu-Raddad L, Chen X-S, Jerse A, Low N, MacLennan CA, Petousis-Harris H, Seib KL, Unemo M, Vincent L, Giersing BK, Gonococcal Vaccine PPC Expert Advisory Group. 2020. Gonococcal vaccines: public health value and preferred product characteristics; report of a WHO global stakeholder consultation, January 2019. Vaccine 38:4362–4373. doi:10.1016/j.vaccine.2020.02.073 32359875 PMC7273195

[9] Prevention CfDCa. 2019. Antibiotic resistance threats in the United States, 2019

[10] WHO. 2012. Global Action Plan to Control the Spread and Impact of Antimicrobial Resistance in Neisseria Gonorrhoeae. Available from: http://whqlibdoc.who.int/publications/2012/9789241503501_eng.pdf

[11] Pacific WHOW. 2011. South East Asian gonococcal antimicrobial surveillance P. surveillance of antibiotic resistance in Neisseria gonorrhoeae in the WHO Western Pacific and South East Asian regions, 2009. Commun Dis Intell Q Rep 35:2–7. 21698977 10.33321/cdi.2011.35.1

[12] World Health Organization. 2015. Antimicrobial resistance draft global action plan on antimicrobial resistance

[13] Centers for Disease Control and Prevention. 2013. Gonorrhea treatment guidelines. Available from: https://www.cdc.gov/nchhstp/newsroom/docs/factsheets/archive/gonorrhea-treatment-guidelines-factsheet.pdf

[14] Centers for Disease Control and Prevention. 2015. Sexually transmitted disease surveillance, 2014.

[15] Alirol E, Wi TE, Bala M, Bazzo ML, Chen X-S, Deal C, Dillon J-A, Kularatne R, Heim J, Hooft van Huijsduijnen R, Hook EW, Lahra MM, Lewis DA, Ndowa F, Shafer WM, Tayler L, Workowski K, Unemo M, Balasegaram M. 2017. Multidrug-resistant gonorrhea: a research and development roadmap to discover new medicines. PLoS Med 14:e1002366. doi:10.1371/journal.pmed.1002366 28746372 PMC5528252

[16] Newman L, Rowley J, Vander Hoorn S, Wijesooriya NS, Unemo M, Low N, Stevens G, Gottlieb S, Kiarie J, Temmerman M. 2015. Global estimates of the prevalence and incidence of four curable sexually transmitted infections in 2012 based on systematic review and global reporting. PLoS One 10:e0143304. doi:10.1371/journal.pone.0143304 26646541 PMC4672879

[17] Fifer H, Natarajan U, Jones L, Alexander S, Hughes G, Golparian D, Unemo M. 2016. Failure of dual antimicrobial therapy in treatment of gonorrhea. N Engl J Med 374:2504–2506. doi:10.1056/NEJMc1512757 27332921

[18] St Cyr S, Barbee L, Workowski KA, Bachmann LH, Pham C, Schlanger K, Torrone E, Weinstock H, Kersh EN, Thorpe P. 2020. Update to CDC’s treatment guidelines for gonococcal infection. MMWR Morb Mortal Wkly Rep 69:1911–1916. doi:10.15585/mmwr.mm6950a6 33332296 PMC7745960

[19] Fleming DT, Wasserheit JN. 1999. From epidemiological synergy to public health policy and practice: the contribution of other sexually transmitted diseases to sexual transmission of HIV infection. Sex Transm Infect 75:3–17. doi:10.1136/sti.75.1.3 10448335 PMC1758168

[20] Trojian TH, Lishnak TS, Heiman D. 2009. Epididymitis and orchitis: an overview. Am Fam Physician 79:583–587. 19378875

[21] Rice PA. 2005. Gonococcal arthritis (disseminated gonococcal infection). Infect Dis Clin North Am 19:853–861. doi:10.1016/j.idc.2005.07.003 16297736

[22] Barr J, Danielsson D. 1971. Septic gonococcal dermatitis. Br Med J 1:482–485. doi:10.1136/bmj.1.5747.482 5101355 PMC1795182

[23] Knapp JS, Holmes KK. 1975. Disseminated gonococcal infections caused by Neisseria gonorrhoeae with unique nutritional requirements. J Infect Dis 132:204–208. doi:10.1093/infdis/132.2.204 125773

[24] Lochner HJ, Maraqa NF. 2018. Sexually transmitted infections in pregnant women: integrating screening and treatment into prenatal care. Paediatr Drugs 20:501–509. doi:10.1007/s40272-018-0310-4 30128814

[25] Humbert MV, Christodoulides M. 2019. Atypical, yet not infrequent, infections with Neisseria species. Pathogens 9:10. doi:10.3390/pathogens9010010 31861867 PMC7168603

[26] Mallika P, Asok T, Faisal H, Aziz S, Tan A, Intan G. 2008. Neonatal conjunctivitis—a review. Malays Fam Physician 3:77–81. 25606121 PMC4170304

[27] Yeu E, Hauswirth S. 2020. A review of the differential diagnosis of acute infectious conjunctivitis: implications for treatment and management. Clin Ophthalmol 14:805–813. doi:10.2147/OPTH.S236571 32210533 PMC7075432

[28] Greenberg L, Diena BB, Ashton FA, Wallace R, Kenny CP, Znamirowski R, Ferrari H, Atkinson J. 1974. Gonococcal vaccine studies in Inuvik. Can J Public Health 65:29–33. 4205640

[29] Greenberg L. 1975. Field trials of a gonococcal vaccine. J Reprod Med 14:34–36. 1089158

[30] Boslego JW, Tramont EC, Chung RC, McChesney DG, Ciak J, Sadoff JC, Piziak MV, Brown JD, Brinton CC, Wood SW, Bryan JR. 1991. Efficacy trial of a parenteral gonococcal pilus vaccine in men. Vaccine 9:154–162. doi:10.1016/0264-410x(91)90147-x 1675029

[31] Kraus SJ, Brown WJ, Arko RJ. 1975. Acquired and natural immunity to gonococcal infection in chimpanzees. J Clin Invest 55:1349–1356. doi:10.1172/JCI108054 805797 PMC301890

[32] Plummer FA, Simonsen JN, Chubb H, Slaney L, Kimata J, Bosire M, Ndinya-Achola JO, Ngugi EN. 1989. Epidemiologic evidence for the development of serovar-specific immunity after gonococcal infection. J Clin Invest 83:1472–1476. doi:10.1172/JCI114040 2496142 PMC303849

[33] Fox KK, Thomas JC, Weiner DH, Davis RH, Sparling PF, Cohen MS. 1999. Longitudinal evaluation of serovar-specific immunity to Neisseria gonorrhoeae. Am J Epidemiol 149:353–358. doi:10.1093/oxfordjournals.aje.a009820 10025478

[34] Schmidt KA, Schneider H, Lindstrom JA, Boslego JW, Warren RA, Van de Verg L, Deal CD, McClain JB, Griffiss JM. 2001. Experimental gonococcal urethritis and reinfection with homologous gonococci in male volunteers. Sex Transm Dis 28:555–564. doi:10.1097/00007435-200110000-00001 11689753

[35] Russell MW, Jerse AE, Gray-Owen SD. 2019. Progress toward a gonococcal vaccine: the way forward. Front Immunol 10:2417. doi:10.3389/fimmu.2019.02417 31681305 PMC6803597

[36] Vincent LR, Jerse AE. 2019. Biological feasibility and importance of a gonorrhea vaccine for global public health. Vaccine 37:7419–7426. doi:10.1016/j.vaccine.2018.02.081 29680200 PMC6892272

[37] Zielke RA, Wierzbicki IH, Baarda BI, Gafken PR, Soge OO, Holmes KK, Jerse AE, Unemo M, Sikora AE. 2016. Proteomics-driven antigen discovery for development of vaccines against gonorrhea. Mol Cell Proteomics 15:2338–2355. doi:10.1074/mcp.M116.058800 27141096 PMC4937508

[38] Zielke RA, Wierzbicki IH, Weber JV, Gafken PR, Sikora AE. 2014. Quantitative proteomics of the Neisseria gonorrhoeae cell envelope and membrane vesicles for the discovery of potential therapeutic targets. Mol Cell Proteomics 13:1299–1317. doi:10.1074/mcp.M113.029538 24607996 PMC4014286

[39] El-Rami FE, Zielke RA, Wi T, Sikora AE, Unemo M. 2019. Quantitative proteomics of the 2016 WHO Neisseria gonorrhoeae reference strains surveys vaccine candidates and antimicrobial resistance determinants. Mol Cell Proteomics 18:127–150. doi:10.1074/mcp.RA118.001125 30352803 PMC6317477

[40] Baarda BI, Zielke RA, Holm AK, Sikora AE. 2021. Comprehensive bioinformatic assessments of the variability of Neisseria gonorrhoeae vaccine candidates. mSphere 6:e00977-20. doi:10.1128/mSphere.00977-20 33536323 PMC7860988

[41] Zielke RA, Le Van A, Baarda BI, Herrera MF, Acosta CJ, Jerse AE, Sikora AE. 2018. SliC is a surface-displayed lipoprotein that is required for the anti-lysozyme strategy during Neisseria gonorrhoeae infection. PLoS Pathog 14:e1007081. doi:10.1371/journal.ppat.1007081 29975784 PMC6033465

[42] Thrane S, Janitzek CM, Matondo S, Resende M, Gustavsson T, de Jongh WA, Clemmensen S, Roeffen W, van de Vegte-Bolmer M, van Gemert GJ, Sauerwein R, Schiller JT, Nielsen MA, Theander TG, Salanti A, Sander AF. 2016. Bacterial superglue enables easy development of efficient virus-like particle based vaccines. J Nanobiotechnology 14:30. doi:10.1186/s12951-016-0181-1 27117585 PMC4847360

[43] Amanna IJ, Carlson NE, Slifka MK. 2007. Duration of humoral immunity to common viral and vaccine antigens. N Engl J Med 357:1903–1915. doi:10.1056/NEJMoa066092 17989383

[44] Schiller J, Lowy D. 2018. Explanations for the high potency of HPV prophylactic vaccines. Vaccine 36:4768–4773. doi:10.1016/j.vaccine.2017.12.079 29325819 PMC6035892

[45] Aves K-L, Goksøyr L, Sander AF. 2020. Advantages and prospects of Tag/Catcher mediated antigen display on capsid-like particle-based vaccines. Viruses 12:185. doi:10.3390/v12020185 32041299 PMC7077247

[46] Mohsen MO, Bachmann MF. 2022. Virus-like particle vaccinology, from bench to bedside. Cell Mol Immunol 19:993–1011. doi:10.1038/s41423-022-00897-8 35962190 PMC9371956

[47] Bachmann MF, Jennings GT. 2010. Vaccine delivery: a matter of size, geometry, kinetics and molecular patterns. Nat Rev Immunol 10:787–796. doi:10.1038/nri2868 20948547

[48] Manolova V, Flace A, Bauer M, Schwarz K, Saudan P, Bachmann MF. 2008. Nanoparticles target distinct dendritic cell populations according to their size. Eur J Immunol 38:1404–1413. doi:10.1002/eji.200737984 18389478

[49] Zabel F, Kündig TM, Bachmann MF. 2013. Virus-induced humoral immunity: on how B cell responses are initiated. Curr Opin Virol 3:357–362. doi:10.1016/j.coviro.2013.05.004 23731601

[50] Jennings GT, Bachmann MF. 2008. The coming of age of virus-like particle vaccines. Biol Chem 389:521–536. doi:10.1515/bc.2008.064 18953718

[51] Kheirvari M, Liu H, Tumban E. 2023. Virus-like particle vaccines and platforms for vaccine development. Viruses 15:1109. doi:10.3390/v15051109 37243195 PMC10223759

[52] Zarreen Simnani F, Singh D, Patel P, Choudhury A, Sinha A, Nandi A, Kumar Samal S, Verma SK, Kumar Panda P. 2023. Nanocarrier vaccine therapeutics for global infectious and chronic diseases. Mater Today 66:371–408. doi:10.1016/j.mattod.2023.04.008

[53] Chackerian B. 2007. Virus-like particles: flexible platforms for vaccine development. Expert Rev Vaccines 6:381–390. doi:10.1586/14760584.6.3.381 17542753

[54] Schödel F, Wirtz R, Peterson D, Hughes J, Warren R, Sadoff J, Milich D. 1994. Immunity to malaria elicited by hybrid hepatitis B virus core particles carrying circumsporozoite protein epitopes. J Exp Med 180:1037–1046. doi:10.1084/jem.180.3.1037 7520465 PMC2191626

[55] Smit MJ, Sander AF, Ariaans M, Fougeroux C, Heinzel C, Fendel R, Esen M, Kremsner PG, Ter Heine R, Wertheim HF, et al.. 2023. First-in-human use of a modular capsid virus-like vaccine platform: an open-label, non-randomised, phase 1 clinical trial of the SARS-CoV-2 vaccine ABNCoV2. Lancet Microbe 4:e140–e148. doi:10.1016/S2666-5247(22)00337-8 36681093 PMC9848408

[56] Zakeri B, Fierer JO, Celik E, Chittock EC, Schwarz-Linek U, Moy VT, Howarth M. 2012. Peptide tag forming a rapid covalent bond to a protein, through engineering a bacterial adhesin. Proc Natl Acad Sci U S A 109:E690–E697. doi:10.1073/pnas.1115485109 22366317 PMC3311370

[57] Escolano A, Gristick HB, Abernathy ME, Merkenschlager J, Gautam R, Oliveira TY, Pai J, West AP, Barnes CO, Cohen AA, et al.. 2019. Immunization expands B cells specific to HIV-1 V3 glycan in mice and macaques. Nature 570:468–473. doi:10.1038/s41586-019-1250-z 31142836 PMC6657810

[58] Leneghan DB, Miura K, Taylor IJ, Li Y, Jin J, Brune KD, Bachmann MF, Howarth M, Long CA, Biswas S. 2017. Nanoassembly routes stimulate conflicting antibody quantity and quality for transmission-blocking malaria vaccines. Sci Rep 7:3811. doi:10.1038/s41598-017-03798-3 28630474 PMC5476561

[59] Palladini A, Thrane S, Janitzek CM, Pihl J, Clemmensen SB, de Jongh WA, Clausen TM, Nicoletti G, Landuzzi L, Penichet ML, Balboni T, Ianzano ML, Giusti V, Theander TG, Nielsen MA, Salanti A, Lollini P-L, Nanni P, Sander AF. 2018. Virus-like particle display of HER2 induces potent anti-cancer responses. Oncoimmunology 7:e1408749. doi:10.1080/2162402X.2017.1408749 29399414 PMC5790387

[60] Fougeroux C, Goksøyr L, Idorn M, Soroka V, Myeni SK, Dagil R, Janitzek CM, Søgaard M, Aves K-L, Horsted EW, et al.. 2021. Capsid-like particles decorated with the SARS-CoV-2 receptor-binding domain elicit strong virus neutralization activity. Nat Commun 12:324. doi:10.1038/s41467-020-20251-8 33436573 PMC7804149

[61] Singh SK, Thrane S, Chourasia BK, Teelen K, Graumans W, Stoter R, van Gemert G-J, van de Vegte-Bolmer MG, Nielsen MA, Salanti A, Sander AF, Sauerwein RW, Jore MM, Theisen M. 2019. Pfs230 and Pfs48/45 fusion proteins elicit strong transmission-blocking antibody responses against Plasmodium falciparum. Front Immunol 10:1256. doi:10.3389/fimmu.2019.01256 31231386 PMC6560166

[62] Janitzek CM, Peabody J, Thrane S, H R Carlsen P, G Theander T, Salanti A, Chackerian B, A Nielsen M, Sander AF. 2019. A proof-of-concept study for the design of a VLP-based combinatorial HPV and placental malaria vaccine. Sci Rep 9:5260. doi:10.1038/s41598-019-41522-5 30918267 PMC6437161

[63] Soongrung T, Mongkorntanyatip K, Peepim T, Jitthamstaporn S, Pitakpolrat P, Kaewamatawong T, Janitzek CM, Thrane S, Sander AF, Jacquet A. 2020. Virus-like particles displaying major HDM allergen Der p 2 for prophylactic allergen immunotherapy. Allergy 75:1232–1236. doi:10.1111/all.14096 31701528

[64] Govasli ML, Diaz Y, Puntervoll P. 2019. Virus-like particle-display of the enterotoxigenic Escherichia coli heat-stable toxoid STh-A14T elicits neutralizing antibodies in mice. Vaccine 37:6405–6414. doi:10.1016/j.vaccine.2019.09.004 31515145

[65] Hu X, Deng Y, Chen X, Zhou Y, Zhang H, Wu H, Yang S, Chen F, Zhou Z, Wang M, Qiu Z, Liao Y. 2017. Immune response of a novel ATR-AP205-001 conjugate anti-hypertensive vaccine. Sci Rep 7:12580. doi:10.1038/s41598-017-12996-y 28974760 PMC5626684

[66] Yenkoidiok-Douti L, Williams AE, Canepa GE, Molina-Cruz A, Barillas-Mury C. 2019. Engineering a virus-like particle as an antigenic platform for a Pfs47-targeted malaria transmission-blocking vaccine. Sci Rep 9:16833. doi:10.1038/s41598-019-53208-z 31727945 PMC6856133

[67] Cohen MS, Cannon JG, Jerse AE, Charniga LM, Isbey SF, Whicker LG. 1994. Human experimentation with Neisseria gonorrhoeae: rationale, methods, and implications for the biology of infection and vaccine development. J Infect Dis 169:532–537. doi:10.1093/infdis/169.3.532 8158024

[68] Wu H, Soler-García AA, Jerse AE. 2009. A strain-specific catalase mutation and mutation of the metal-binding transporter gene mntC attenuate Neisseria gonorrhoeae in vivo but not by increasing susceptibility to oxidative killing by phagocytes. Infect Immun 77:1091–1102. doi:10.1128/IAI.00825-08 19114548 PMC2643649

[69] Kellogg DS, Peacock WL, Deacon WE, Brown L, Pirkle DI. 1963. Neisseria gonorrhoeae. I. Virulence genetically linked to clonal variation. J Bacteriol 85:1274–1279. doi:10.1128/jb.85.6.1274-1279.1963 14047217 PMC278328

[70] Spence JM, Wright L, Clark VL. 2008. Laboratory maintenance of Neisseria gonorrhoeae. Curr Protoc Microbiol Chapter 4:Unit 4A.1. doi:10.1002/9780471729259.mc04a01s8 18770539

[71] Studier FW, Moffatt BA. 1986. Use of bacteriophage T7 RNA polymerase to direct selective high-level expression of cloned genes. J Mol Biol 189:113–130. doi:10.1016/0022-2836(86)90385-2 3537305

[72] Sikora AE, Gomez C, Le Van A, Baarda BI, Darnell S, Martinez FG, Zielke RA, Bonventre JA, Jerse AE. 2020. A novel gonorrhea vaccine composed of MetQ lipoprotein formulated with CpG shortens experimental murine infection. Vaccine 38:8175–8184. doi:10.1016/j.vaccine.2020.10.077 33162204 PMC7704770

[73] Gulati S, Beurskens FJ, de Kreuk B-J, Roza M, Zheng B, DeOliveira RB, Shaughnessy J, Nowak NA, Taylor RP, Botto M, He X, Ingalls RR, Woodruff TM, Song W-C, Schuurman J, Rice PA, Ram S. 2019. Complement alone drives efficacy of a chimeric antigonococcal monoclonal antibody. PLoS Biol 17:e3000323. doi:10.1371/journal.pbio.3000323 31216278 PMC6602280

[74] Gulati S, Pennington MW, Czerwinski A, Carter D, Zheng B, Nowak NA, DeOliveira RB, Shaughnessy J, Reed GW, Ram S, Rice PA. 2019. Preclinical efficacy of a lipooligosaccharide peptide mimic candidate gonococcal vaccine. mBio 10:e02552-19. doi:10.1128/mBio.02552-19 31690678 PMC6831779

[75] Sikora AE, Wierzbicki IH, Zielke RA, Ryner RF, Korotkov KV, Buchanan SK, Noinaj N. 2018. Structural and functional insights into the role of BamD and BamE within the beta-barrel assembly machinery in Neisseria gonorrhoeae. J Biol Chem 293:1106–1119. doi:10.1074/jbc.RA117.000437 29229778 PMC5787791

[76] Karch CP, Burkhard P. 2016. Vaccine technologies: from whole organisms to rationally designed protein assemblies. Biochem Pharmacol 120:1–14. doi:10.1016/j.bcp.2016.05.001 27157411 PMC5079805

[77] Calabro S, Tritto E, Pezzotti A, Taccone M, Muzzi A, Bertholet S, De Gregorio E, O’Hagan DT, Baudner B, Seubert A. 2013. The adjuvant effect of MF59 is due to the oil-in-water emulsion formulation, none of the individual components induce a comparable adjuvant effect. Vaccine 31:3363–3369. doi:10.1016/j.vaccine.2013.05.007 23684834

[78] Giuntini S, Reason DC, Granoff DM. 2012. Combined roles of human IgG subclass, alternative complement pathway activation, and epitope density in the bactericidal activity of antibodies to meningococcal factor h binding protein. Infect Immun 80:187–194. doi:10.1128/IAI.05956-11 22064712 PMC3255668

[79] Ragland SA, Humbert MV, Christodoulides M, Criss AK. 2018. Neisseria gonorrhoeae employs two protein inhibitors to evade killing by human lysozyme. PLoS Pathog 14:e1007080. doi:10.1371/journal.ppat.1007080 29975775 PMC6033460

[80] Humbert MV, Awanye AM, Lian L-Y, Derrick JP, Christodoulides M. 2017. Structure of the Neisseria adhesin complex protein (ACP) and its role as a novel lysozyme inhibitor. PLoS Pathog 13:e1006448. doi:10.1371/journal.ppat.1006448 28662181 PMC5507604

[81] Sikora AE, Gomez C, Le Van A, Baarda BI, Darnell S, Martinez FG, Zielke RA, Bonventre JA, Jerse AE. 2020. A novel gonorrhea vaccine composed of MetQ lipoprotein formulated with CpG shortens experimental murine infection. bioRxiv:20200619161646. doi: 10.1101/2020.06.19.161646 10.1016/j.vaccine.2020.10.077 PMC7704770 33162204

[82] Zhu W, Chen C-J, Thomas CE, Anderson JE, Jerse AE, Sparling PF. 2011. Vaccines for gonorrhea: can we rise to the challenge? Front Microbiol 2:124. doi:10.3389/fmicb.2011.00124 21687431 PMC3109613

[83] Gulati S, Zheng B, Reed GW, Su X, Cox AD, St Michael F, Stupak J, Lewis LA, Ram S, Rice PA. 2013. Immunization against a saccharide epitope accelerates clearance of experimental gonococcal infection. PLoS Pathog 9:e1003559. doi:10.1371/journal.ppat.1003559 24009500 PMC3757034

[84] Amanda DeRocco HFS, Sempowski G, Ventevogel M, Jerse AE. 2014. Development of MtrE, the outer membrane channel of the MtrCDE and FarAB,MtrE active efflux pump systems, as a gonorrhea vaccine. International Pathogenic Neisseria Conference.

[85] Liu Y, Perez J, Hammer LA, Gallagher HC, De Jesus M, Egilmez NK, Russell MW. 2018. Intravaginal administration of interleukin 12 during genital gonococcal infection in mice induces immunity to heterologous strains of Neisseria gonorrhoeae. mSphere 3:e00421-17. doi:10.1128/mSphere.00421-17 29404418 PMC5793040

[86] Zhu W, Thomas CE, Chen C-J, Van Dam CN, Johnston RE, Davis NL, Sparling PF. 2005. Comparison of immune responses to gonococcal PorB delivered as outer membrane vesicles, recombinant protein, or Venezuelan equine encephalitis virus replicon particles. Infect Immun 73:7558–7568. doi:10.1128/IAI.73.11.7558-7568.2005 16239559 PMC1273881

[87] Liu Y, Hammer LA, Daamen J, Stork M, Egilmez NK, Russell MW. 2023. Microencapsulated IL-12 drives genital tract immune responses to intranasal gonococcal outer membrane vesicle vaccine and induces resistance to vaginal infection with diverse strains of Neisseria gonorrhoeae. mSphere 8:e0038822. doi:10.1128/msphere.00388-22 36537786 PMC9942569

[88] Li G, Jiao H, Jiang G, Wang J, Zhu L, Xie R, Yan H, Chen H, Ji M. 2011. Neisseria gonorrhoeae NspA induces specific bactericidal and opsonic antibodies in mice. Clin Vaccine Immunol 18:1817–1822. doi:10.1128/CVI.05245-11 21918113 PMC3209019

[89] Haneberg B, Dalseg R, Wedege E, Høiby EA, Haugen IL, Oftung F, Andersen SR, Naess LM, Aase A, Michaelsen TE, Holst J. 1998. Intranasal administration of a meningococcal outer membrane vesicle vaccine induces persistent local mucosal antibodies and serum antibodies with strong bactericidal activity in humans. Infect Immun 66:1334–1341. doi:10.1128/IAI.66.4.1334-1341.1998 9529050 PMC108057

[90] Chackerian B, Lenz P, Lowy DR, Schiller JT. 2002. Determinants of autoantibody induction by conjugated papillomavirus virus-like particles. J Immunol 169:6120–6126. doi:10.4049/jimmunol.169.11.6120 12444114

[91] Safaeian M, Porras C, Pan Y, Kreimer A, Schiller JT, Gonzalez P, Lowy DR, Wacholder S, Schiffman M, Rodriguez AC, Herrero R, Kemp T, Shelton G, Quint W, van Doorn L-J, Hildesheim A, Pinto LA, CVT Group. 2013. Durable antibody responses following one dose of the bivalent human papillomavirus L1 virus-like particle vaccine in the Costa Rica vaccine trial. Cancer Prev Res (Phila) 6:1242–1250. doi:10.1158/1940-6207.CAPR-13-0203 24189371 PMC7605443

[92] Adkins JC, Wagstaff AJ. 1998. Recombinant hepatitis B vaccine: a review of its immunogenicity and protective efficacy against hepatitis B. BioDrugs 10:137–158. doi:10.2165/00063030-199810020-00005 18020591

[93] Dertzbaugh MT. 1998. Genetically engineered vaccines: an overview. Plasmid 39:100–113. doi:10.1006/plas.1997.1329 9514708

[94] Cao Y, Bing Z, Guan S, Zhang Z, Wang X. 2018. Development of new hepatitis E vaccines. Hum Vaccin Immunother 14:2254–2262. doi:10.1080/21645515.2018.1469591 29708836 PMC6183316

[95] McLemore MR. 2006. Gardasil: introducing the new human papillomavirus vaccine. Clin J Oncol Nurs 10:559–560. doi:10.1188/06.CJON.559-560 17063609

[96] Szarewski A. 2010. HPV vaccine: cervarix. Expert Opin Biol Ther 10:477–487. doi:10.1517/14712591003601944 20132062

[97] Donaldson B, Lateef Z, Walker GF, Young SL, Ward VK. 2018. Virus-like particle vaccines: immunology and formulation for clinical translation. Expert Rev Vaccines 17:833–849. doi:10.1080/14760584.2018.1516552 30173619 PMC7103734

[98] Janitzek CM, Carlsen PHR, Thrane S, Khanna VM, Jakob V, Barnier-Quer C, Collin N, Theander TG, Salanti A, Nielsen MA, Sander AF. 2021. The immunogenicity of capsid-like particle vaccines in combination with different adjuvants using different routes of administration. Vaccines (Basel) 9:131. doi:10.3390/vaccines9020131 33562114 PMC7915698

[99] Tissot AC, Renhofa R, Schmitz N, Cielens I, Meijerink E, Ose V, Jennings GT, Saudan P, Pumpens P, Bachmann MF. 2010. Versatile virus-like particle carrier for epitope based vaccines. PLoS One 5:e9809. doi:10.1371/journal.pone.0009809 20352110 PMC2843720

[100] van den Worm SHE, Koning RI, Warmenhoven HJ, Koerten HK, van Duin J. 2006. Cryo electron microscopy reconstructions of the Leviviridae unveil the densest icosahedral RNA packing possible. J Mol Biol 363:858–865. doi:10.1016/j.jmb.2006.08.053 16989861

[101] Shishovs M, Rumnieks J, Diebolder C, Jaudzems K, Andreas LB, Stanek J, Kazaks A, Kotelovica S, Akopjana I, Pintacuda G, Koning RI, Tars K. 2016. Structure of AP205 coat protein reveals circular permutation in ssRNA bacteriophages. J Mol Biol 428:4267–4279. doi:10.1016/j.jmb.2016.08.025 27591890

[102] Maurakis SA, Cornelissen CN. 2022. Recent progress towards a gonococcal vaccine. Front Cell Infect Microbiol 12:881392. doi:10.3389/fcimb.2022.881392 35480233 PMC9038166

[103] Scheiblhofer S, Laimer J, Machado Y, Weiss R, Thalhamer J. 2017. Influence of protein fold stability on immunogenicity and its implications for vaccine design. Expert Rev Vaccines 16:479–489. doi:10.1080/14760584.2017.1306441 28290225 PMC5490637

[104] Liu Y, Hammer LA, Liu W, Hobbs MM, Zielke RA, Sikora AE, Jerse AE, Egilmez NK, Russell MW. 2017. Experimental vaccine induces Th1-driven immune responses and resistance to Neisseria gonorrhoeae infection in a murine model. Mucosal Immunol 10:1594–1608. doi:10.1038/mi.2017.11 28272393 PMC5591041

[105] Zhu W, Thomas CE, Sparling PF. 2004. DNA immunization of mice with a plasmid encoding Neisseria gonorrhea PorB protein by intramuscular injection and epidermal particle bombardment. Vaccine 22:660–669. doi:10.1016/j.vaccine.2003.08.036 14741158

[106] Lewis LA, Gulati S, Zelek WM, Morgan BP, Song W-C, Zheng B, Nowak N, DeOliveira RB, Sanchez B, DeSouza Silva L, Schuurman J, Beurskens F, Ram S, Rice PA. 2022. Efficacy of an experimental gonococcal lipooligosaccharide mimitope vaccine requires terminal complement. J Infect Dis 225:1861–1864. doi:10.1093/infdis/jiab630 34971376 PMC9113499

[107] Baarda BI, Martinez FG, Sikora AE. 2018. Proteomics, bioinformatics and structure-function antigen mining for gonorrhea vaccines. Front Immunol 9:2793. doi:10.3389/fimmu.2018.02793 30564232 PMC6288298

[108] Plotkin SA. 2010. Correlates of protection induced by vaccination. Clin Vaccine Immunol 17:1055–1065. doi:10.1128/CVI.00131-10 20463105 PMC2897268

[109] Johansson M, Lycke NY. 2003. Immunology of the human genital tract. Curr Opin Infect Dis 16:43–49. doi:10.1097/00001432-200302000-00008 12821829

[110] Li Z, Palaniyandi S, Zeng R, Tuo W, Roopenian DC, Zhu X. 2011. Transfer of IgG in the female genital tract by MHC class I-related neonatal Fc receptor (FcRn) confers protective immunity to vaginal infection. Proc Natl Acad Sci U S A 108:4388–4393. doi:10.1073/pnas.1012861108 21368166 PMC3060240

