# Supplementary material for: Development of a Tag/Catcher-mediated capsid virus-like particle vaccine presenting the conserved Neisseria gonorrhoeae SliC antigen that blocks human lysozyme
Source: Infect Immun. 2023 Nov 2;91(12):e00245-23. doi: 10.1128/iai.00245-23 (PMC10715030; doi:10.1128/iai.00245-23)
Supplement: Figure S1 — Lysozyme assay. [file iai.00245-23-s0001.pdf]

Supplemental Material

Supplemental Figures

Figure S1. Assessment of SliC and ACP activity against human lysozyme. (**A**) To determine if addition of SpyTag affects the SliC-mediated inhibition of c-type human, samples containing 2.5 µM human lysozyme (HL) were incubated with increasing concentrations of N-SliC (0-5 µM) for 30 min at 37°C. The controls contained HL alone. After incubation, the reaction was initiated by addition of DQ lysozyme substrate. The reaction was monitored for 20 min at excitation and emission wavelengths of 485 nm and 530 nm, respectively. (**B**, **C**, **D**) To examine if immunization with SliC/ACP elicit antigen function blocking antibodies, SliC/ACP were incubated with pooled sera from rabbits (**B** and **C**), or mice (**B** and **D**) immunized and control groups (1:10 v/v), as designated, for 30 min and the lysozyme assays were carried out as described above.

A.

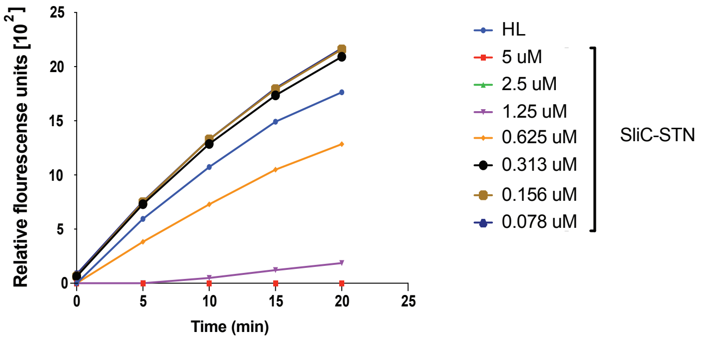
Relative flourescense units [10 2]
Time (min)
0
5
10
15
20
25
HL
5 uM
2.5 uM
1.25 uM
0.625 uM
0.313 uM
0.156 uM
0.078 uM
SliC-STN

B.

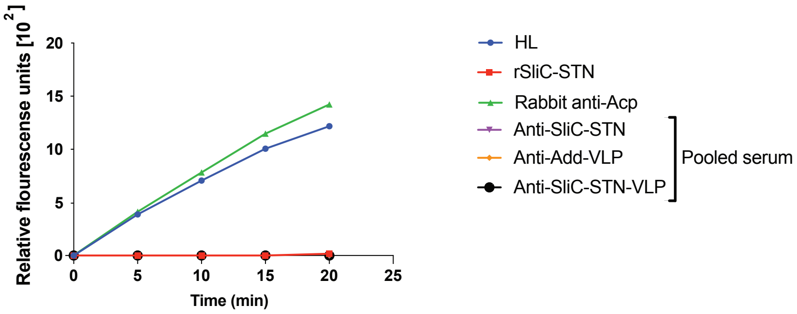
Relative flourescense units [10 2]
Time (min)
0
5
10
15
20
25
HL
rSliC-STN
Rabbit anti-Acp
Anti-SliC-STN
Anti-Add-VLP
Anti-SliC-STN-VLP
Pooled serum

C.

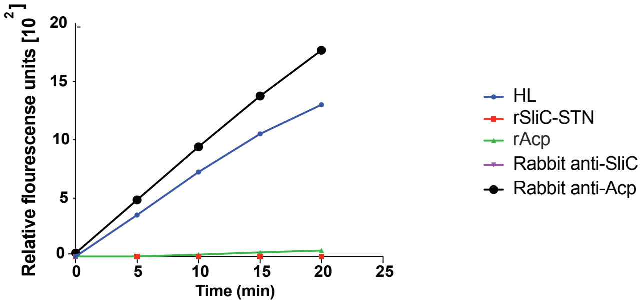
Relative flourescense units [10 2]
Time (min)
0
5
10
15
20
25
HL
rSliC-STN
rAcp
Rabbit anti-SliC
Rabbit anti-Acp

D.

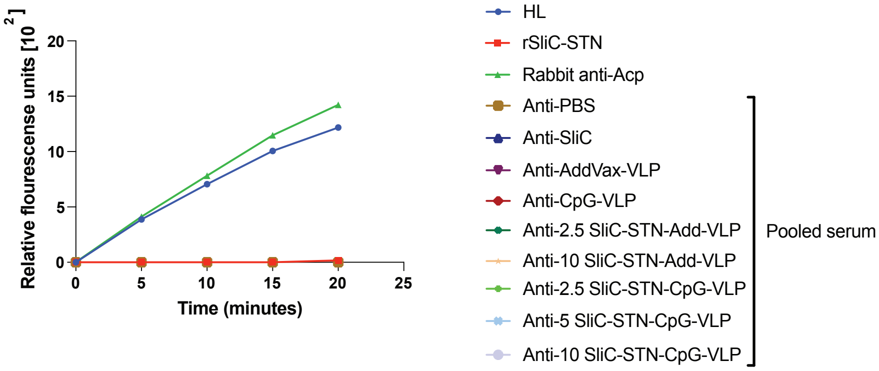
Relative flourescense units [10 2]
Time (minutes)
0
5
10
15
20
25
HL
rSliC-STN
Rabbit anti-Acp
Anti-PBS
Anti-SliC
Anti-AddVax-VLP
Anti-CpG-VLP
Anti-2.5 SliC-STN-Add-VLP
Anti-10 SliC-STN-Add-VLP
Anti-2.5 SliC-STN-CpG-VLP
Anti-5 SliC-STN-CpG-VLP
Anti-10 SliC-STN-CpG-VLP
Pooled serum
